# The super-healing MRL strain promotes muscle growth in muscular dystrophy through a regenerative extracellular matrix

**DOI:** 10.1172/jci.insight.173246

**Published:** 2024-01-04

**Authors:** Joseph G. O’Brien, Alexander B. Willis, Ashlee M. Long, Jason Kwon, GaHyun Lee, Frank W. Li, Patrick G.T. Page, Andy H. Vo, Michele Hadhazy, Melissa J. Spencer, Rachelle H. Crosbie, Alexis R. Demonbreun, Elizabeth M. McNally

**Affiliations:** 1 Center for Genetic Medicine, Northwestern University Feinberg School of Medicine, Chicago, Illinois, USA.; 2 Department of Neurology, David Geffen School of Medicine, UCLA, Los Angeles, California, USA.; 3 Department of Integrative Biology and Physiology, Department of Neurology, David Geffen School of Medicine, UCLA, Los Angeles, California, USA.; 4 Department of Pharmacology, Northwestern University Feinberg School of Medicine, Chicago, Illinois, USA.

**Keywords:** Muscle Biology, Stem cells, Extracellular matrix, Fibrosis

## Abstract

The Murphy Roths Large (MRL) mouse strain has “super-healing” properties that enhance recovery from injury. In mice, the DBA/2J strain intensifies many aspects of muscular dystrophy, so we evaluated the ability of the MRL strain to suppress muscular dystrophy in the *Sgcg*-null mouse model of limb girdle muscular dystrophy. A comparative analysis of *Sgcg*-null mice in the DBA/2J versus MRL strains showed greater myofiber regeneration, with reduced structural degradation of muscle in the MRL strain. Transcriptomic profiling of dystrophic muscle indicated strain-dependent expression of extracellular matrix (ECM) and TGF-β signaling genes. To investigate the MRL ECM, cellular components were removed from dystrophic muscle sections to generate decellularized myoscaffolds. Decellularized myoscaffolds from dystrophic mice in the protective MRL strain had significantly less deposition of collagen and matrix-bound TGF-β1 and TGF-β3 throughout the matrix. Dystrophic myoscaffolds from the MRL background, but not the DBA/2J background, were enriched in myokines like IGF-1 and IL-6. C2C12 myoblasts seeded onto decellularized matrices from *Sgcg^–/–^* MRL and *Sgcg^–/–^* DBA/2J muscles showed the MRL background induced greater myoblast differentiation compared with dystrophic DBA/2J myoscaffolds. Thus, the MRL background imparts its effect through a highly regenerative ECM, which is active even in muscular dystrophy.

## Abstract 2

<p>The extracellular matrix of the super-healing MRL mouse strain harbors regenerative myokines that improve skeletal muscle growth and function in muscular dystrophy.</p>

## Graphical abstract

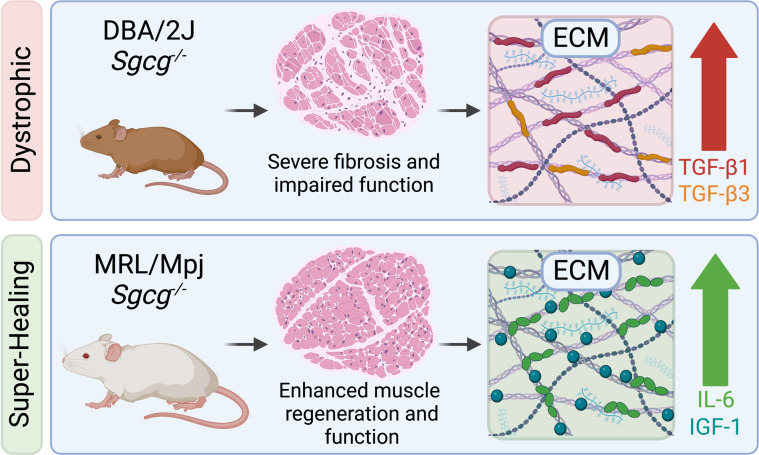

## Introduction

Genetic disruption of the dystrophin glycoprotein complex (DGC) causes muscular dystrophy in humans, and multiple mouse models bear mutations in DGC-encoding genes (1, 2). A shared molecular pathology in these disorders is the interrupted connection of the intracellular cytoskeleton from the plasma membrane and the extracellular matrix (ECM) (3–5). Loss-of-function mutations that disrupt the sarcoglycan subunits within the DGC lead to limb girdle muscular dystrophy (LGMD) (6). The severity of LGMD caused by mutations in *SGCG*, the gene encoding the γ-sarcoglycan subunit, is highly variable (7). This phenotypic variability has been recapitulated with the generation of *Sgcg*-null (*Sgcg^–/–^*) mice and the introduction of this allele into multiple different background strains (8, 9). The DBA/2J (D2) strain intensifies many of the features of muscular dystrophy, including muscle plasma membrane instability, fibrosis, and functional decline (9, 10). For the *Sgcg* mutation, the C57BL/6J (B6) genetic background, like the closely related C57BL/10, is less severe than D2 but still produces evident fibrosis and impaired muscle function (9, 11, 12), which can be corrected through gene replacement therapy (13, 14).

The Murphy Roths Large (MRL) mouse strain has been studied for its ability to suppress fibrosis and accelerate wound healing in many physiological settings (15, 16). First discovered for its ability to form “scarless” wound healing, the MRL strain is thought to impart both enhanced regeneration and repair (15, 17). In muscle injury, a comparative analysis of the MRL and B6 strains after cardiotoxin injury showed rapid reduction of wound size and progenitor muscle cell activation in the MRL strain (18). Enhanced recovery after injury was accompanied by increased expression of antioxidants in the MRL background (18). It was also shown that muscle-derived stem cells isolated from the MRL strain have enhanced expression of *Myod1*, *Myog*, and *Pax7* transcripts in response to injury, and hypoxia-inducible factor 1α (HIF-1α) contributed to the increase in *Pax7* expression (19).

The ECM mediates muscle regeneration and repair through its structural proteins as well as the smaller cytokine proteins embedded within these structural elements (20, 21). Transforming growth factor β (TGF-β) is increased in both acute and chronic muscle injury, which in turn, further upregulates collagen and fibronectin (22–24). Mouse models of muscular dystrophy in the D2 strain have markedly elevated TGF-β proteins, in part mediated by polymorphisms in the latent TGF-β binding protein 4 (*Ltbp4*) as well as a feedback loop with osteopontin (*Spp1*) (25). The link between ECM composition and disease progression has prompted therapeutic development targeting the ECM (26). A recently described method permits evaluation of decellularized dystrophic muscle in an on-slide format (27). The generation of these “myoscaffolds” leaves a biologically intact ECM that retains cross-sectional spatial information of the dystrophic muscle tissue (27). Using this optimized decellularization method, the ECM protein laminin was observed to undergo remodeling by skeletal muscle progenitor cells in less severely scarred areas of dystrophic muscle, highlighting the importance of specific ECM proteins in mediating cellular regeneration (27).

The MRL background was previously observed to partially mitigate the severity of fibrosis that characterizes *Sgcg^–/–^* mice in the D2 background (*Sgcg*-D2) (28). The prior breeding strategy evaluated the effect of having a 50% contribution of the MRL background and found the hybrid *Sgcg*-MRL/D2 background demonstrated a significant decrease of fibrosis in skeletal and cardiac muscle (28). The *Sgcg*-MRL/D2 hybrid background showed some evidence for enhanced regeneration, with increased embryonic myosin heavy chain expression and a higher proportion of centralized nuclei in several muscle groups compared with the *Sgcg*-D2 background. However, this hybrid background only partly improved some aspects of *Sgcg*-mediated muscular dystrophy. We have extended these findings by generating the *Sgcg*-null allele on a full MRL background, referred to as *Sgcg*-MRL. We elected to carry out this cross, beginning with *Sgcg*-D2 mice to assess whether the MRL background was sufficient to correct the severely fibrotic features induced by the D2 strain. The histological, functional, and molecular signatures of *Sgcg*-MRL mice were enhanced in comparison with *Sgcg*-D2 mice, demonstrating that the MRL background produces sustained effects over the repeated degeneration that characterizes muscular dystrophy. Prompted by a transcriptomic profile of reduced fibrosis, we interrogated decellularized myoscaffolds to investigate how the super-healing MRL background modified the ECM. We found that C2C12 myoblasts seeded onto the MRL ECM adopted mobility and morphology features of enhanced regeneration. These studies show that the super-healing properties of the MRL background derive from having a regenerative ECM.

## Results

### Reduced muscle fibrosis and enhanced respiratory muscle function in the super-healing MRL background strain.

*Sgcg^–/–^* mice lack the dystrophin-associated protein, γ-sarcoglycan, and this model was previously shown to display a more intense muscular dystrophy process when present in the D2 background strain, which produced greater muscle fibrosis and correspondingly weaker muscles (9). We queried whether the MRL background could suppress this more severe muscular dystrophy phenotype by backcrossing the *Sgcg^–/–^* allele into the MRL strain over 10 generations. Backcrosses used both male and female *Sgcg^+/–^* heterozygous animals. After 10 generations, heterozygous *Sgcg^+/–^* animals were intercrossed to generate homozygous *Sgcg^–/–^* animals. The nature of this breeding generates the *Sgcg* exon 2–deleted null allele in the context of the MRL genome.

**Figure 1:**
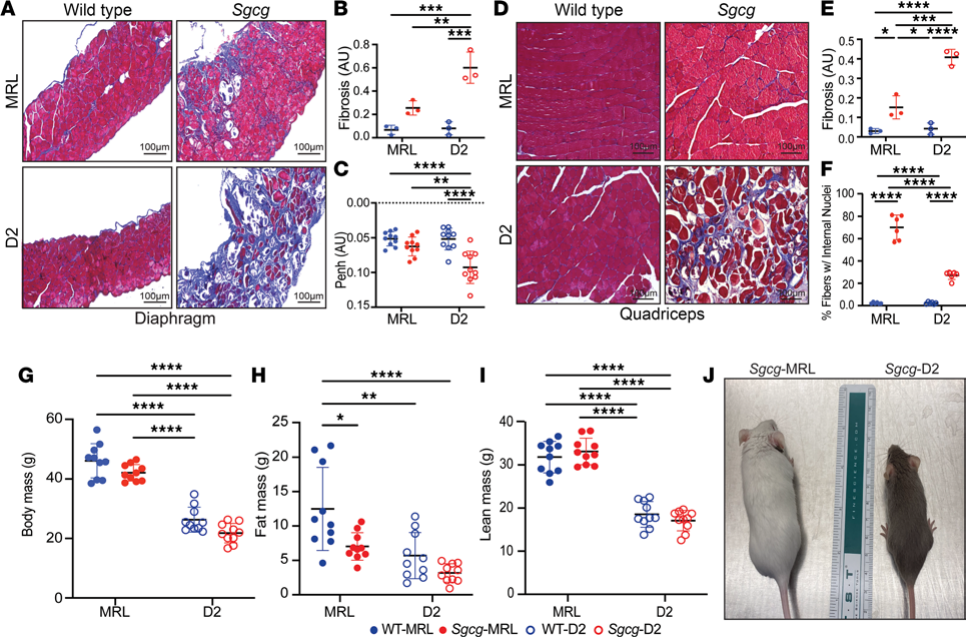
The MRL background reduced fibrosis in the muscles of *Sgcg^–/–^* mice. Comparative phenotypic assessment of skeletal muscle in the *Sgcg*-MRL, *Sgcg*-D2, WT-MRL, and WT-D2 strains was conducted over 20 weeks, including 5 males and 5 females. (**A**) Representative Masson’s trichrome staining of the diaphragm muscles (20 weeks) showed the MRL background significantly improved fibrosis in *Sgcg^–/–^* mice. (**B**) Reduction of mean fibrosis (blue staining) relative to total muscle area in the MRL background (WT-MRL 0.03, *Sgcg*-MRL 0.15, WT-D2 0.04, and *Sgcg*-D2 0.41 AU). (**C**) Average Penh, a measure of impaired respiratory function, was corrected by the MRL background (WT-MRL 0.05, *Sgcg*-MRL 0.06, WT-D2 0.05, and *Sgcg*-D2 0.09 AU). (**D**) Representative images of the quadriceps muscles with reduced fibrosis in *Sgcg*-MRL mice. (**E**) Reduction of mean fibrosis in the quadriceps muscles relative to total muscle area in the MRL strain (WT-MRL 0.07, *Sgcg*-MRL 0.26, WT-D2 0.08, and *Sgcg*-D2 0.62 AU). (**F**) Mean percentage of fibers containing centralized nuclei was increased in the *Sgcg*-MRL mice (WT-MRL 2%, *Sgcg*-MRL 70%, WT-D2 2%, *Sgcg*-D2 27%). (**G**) Body mass was increased in the MRL background. (**H**) Mean fat mass was highest in the WT-MRL cohort (WT-MRL 12.5, *Sgcg*-MRL 7, WT-D2 5.7, and *Sgcg*-D2 3.2 g). (**I**) Mean lean mass was significantly higher in both MRL models compared with the D2 models (WT-MRL 31.8, *Sgcg*-MRL 18.5, WT-D2 33.1, and *Sgcg*-D2 17.1 g). (**J**) Representative image depicting the size variation between the *Sgcg*-MRL and *Sgcg*-D2 mice. Scale bars (**B** and **D**): 100 μm. Data are presented as mean ± SD. *P < 0.05; **P < 0.01; ***P < 0.001; ****P < 0.0001 by 2-way ANOVA with Tukey’s multiple-comparison test.

Terminal evaluation of *Sgcg^–/–^* muscles at 20 weeks of age was used to assess outcome. We compared the respective wild-type (WT) from the MRL and D2 backgrounds (referred to at WT-MRL and WT-D2, respectively) alongside *Sgcg^–/–^* mice from the MRL and D2 backgrounds (referred to as *Sgcg*-MRL and *Sgcg*-D2 respectively). The cohorts were age and sex matched, with of 5 male and 5 females. Masson’s trichrome staining of the diaphragm muscle from 20-week-old male mice demonstrated markedly reduced fibrosis in *Sgcg*-MRL muscle compared with *Sgcg*-D2 (Figure 1A). The *Sgcg*-D2 model had increased accumulation of ECM fibrosis in the diaphragm muscle (Figure 1B), with a corresponding decrease in function. *Sgcg*-D2 mice demonstrated impaired respiratory function at 10 weeks of age as evidenced by elevated Penh, a composite plethysmography measure (29). *Sgcg*-MRL mice had Penh values indistinguishable from WT-MRL mice and WT-D2 mice, consistent with the reduction in fibrosis imparted by the MRL strain improving muscle function (Figure 1C). We also evaluated quadriceps muscles and identified significantly reduced pathological features in *Sgcg-*MRL compared with *Sgcg*-D2 mice (Figure 1D). Fibrosis was reduced in the *Sgcg*-MRL quadriceps muscles compared with *Sgcg*-D2 mice (Figure 1E). There were significantly more internal myonuclei in the muscles of the *Sgcg*-MRL mice (Figure 1F), consistent with an enhanced regenerative response in MRL skeletal muscle. Serum creatine kinase (CK) was markedly elevated in *Sgcg*-MRL and *Sgcg*-D2 mice compared with strain-matched WT mice (Supplemental Figure 1A; supplemental material available online with this article; https://doi.org/10.1172/jci.insight.173246DS1). However, by 20 weeks, serum CK declined in *Sgcg*-D2 mice, correlating with the comparative wasting seen in animals at this age (Supplemental Figure 1, B–D), and similar to what is seen in patients with more advanced muscular dystrophy who have reduced muscle mass (30). Elevated CK in *Sgcg*-MRL mice, accompanied by reduced fibrosis, points to a role for enhanced regeneration and repair in the MRL background.

At 20 weeks of age, total body mass showed a strong strain dependency, as WT-MRL mice were much larger than WT-D2 mice (Figure 1G). Body composition analysis by NMR assessed fat and lean contributions of body mass (Figure 1, H and I, respectively). In the MRL strain, there were sex-specific differences in body mass composition. Although total body mass was similar between male and female WT-MRL mice (Supplemental Figure 2A), female WT-MRL mice had significantly increased fat mass and correspondingly reduced lean mass compared with male WT-MRL mice (Supplemental Figure 2, B and C). This sex-specific body mass composition was absent in *Sgcg*-MRL mice, where male and female dystrophic mice had similar fat-to-lean mass ratios. In D2 mice, sex-specific differences in body mass were less evident and a trend toward reduced lean mass was seen for *Sgcg*-D2 males relative to WT-D2 (Supplemental Figure 2D). By 20 weeks of age, a stage when *Sgcg*-D2 mice display kyphoscoliosis and wasting in both sexes relative to their WT counterparts, *Sgcg-*MRL mice appeared outwardly indistinguishable from their WT counterparts (Figure 1J).

### Larger muscles in MRL mice compared with D2 mice.

**Figure 2:**
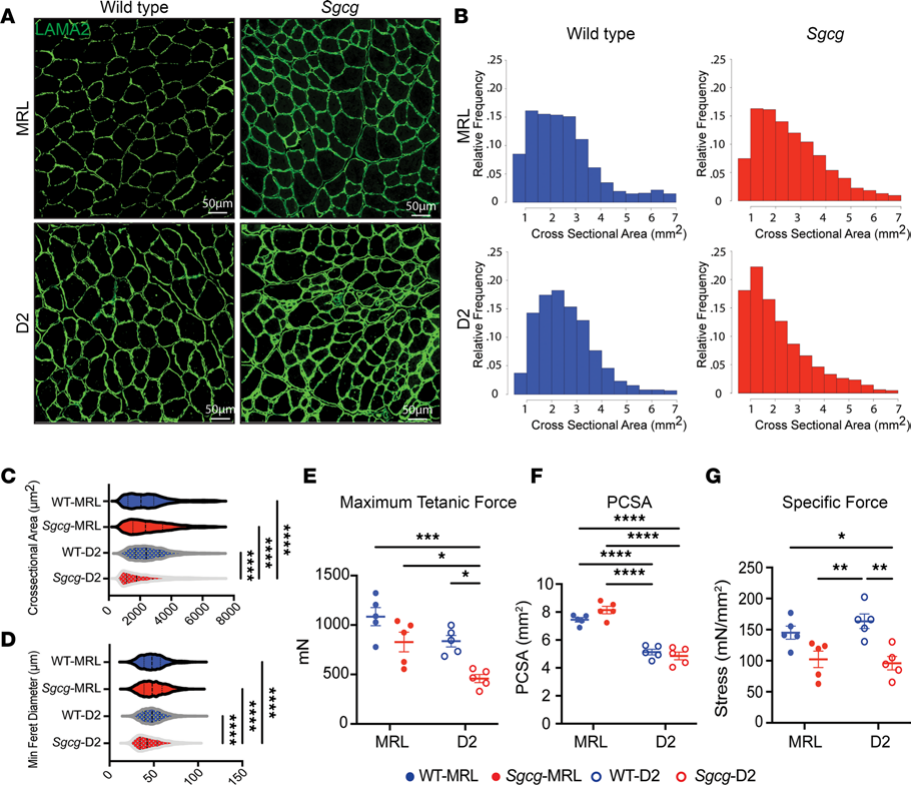
The MRL strain increased myofiber size and maximum tetanic force in *Sgcg*^–/–^ mice. TA muscle was analyzed at 20 weeks. (**A**) Representative immunofluorescence microscopy (IFM) images showed larger myofibers in the TA of the *Sgcg*-MRL stained for laminin-α2 (LAMA2). Scale bars: 50 μm. (**B**) Cross-sectional area (CSA) of myofibers was shifted rightward in *Sgcg-*MRL muscle. (**C**) CSA was increased in the MRL background (WT-MRL 2544, *Sgcg*-MRL 2619, WT-D2 2560, and *Sgcg*-D2 2154 μm^2^). (**D**) MRL background increased the minimum Feret diameter in the *Sgcg^–/–^* muscle (WT-MRL 49.1, *Sgcg*-MRL 49.6, WT-D2 49.6, and *Sgcg*-D2 45.1 μm). (**E**) Force measurements from male TA muscles showing maximum tetanic force was significantly reduced in the *Sgcg*-D2 strains compared with all other strains (WT-MRL 1085, *Sgcg*-MRL 828.5, WT-D2 836.3, and *Sgcg*-D2 458.7 mN). (**F**) Physiological CSA (PCSA) was smaller in D2 compared with MRL muscle for both *Sgcg^–/–^* and WT (WT-MRL 7.44, *Sgcg*-MRL 8.14, WT-D2 5.1, and *Sgcg*-D2 4.85 mm^2^). (**G**) Specific force was reduced for *Sgcg^–/–^* compared with WT in the D2 strain but not the MRL strain (WT-MRL 145, *Sgcg*-MRL 102.3, WT-D2 163.5, and *Sgcg*-D2 96.0 mN/mm^2^). Data are presented as mean ± SD. *P < 0.05; **P < 0.01; ***P < 0.001; ****P < 0.0001 by 2-way ANOVA with Tukey’s multiple-comparison test.

The increase in centrally nucleated myofibers in *Sgcg*-MRL muscle compared with *Sgcg*-D2 muscle suggests the MRL background promotes growth and regeneration. To further investigate muscle morphometric features, we evaluated the tibialis anterior (TA) muscles, as these muscles are also suitable for physiological assessment. Laminin-α2 (LAMA2) staining was used to outline myofibers and assess fiber size and myofiber cross-sectional area (CSA) (Figure 2, A and B). A higher proportion of smaller myofibers was present in the TA muscles from *Sgcg*-D2 mice, and these small myofibers were not apparent in TA muscles from *Sgcg*-MRL mice. Further analyses of the CSA and minimum Feret diameter demonstrated a decrease in muscle fiber size in the *Sgcg*-D2 mice compared with fibers from WT-D2 and *Sgcg*-MRL mice (Figure 2, C and D), and these findings are consistent with the impaired growth of *Sgcg*-D2 muscle compared with the proregenerative environment of the MRL background strain. Fiber type composition analysis showed that the TA muscles from the MRL mouse strain contained a higher proportion of fast twitch (type IIB) muscle fibers compared with D2 muscles, and this was apparent in both WT-MRL and *Sgcg*-MRL mice (Supplemental Figure 3A). These results were further validated by counting cryosection fibers, which confirmed the higher proportion of type IIB fibers in the MRL background (Supplemental Figure 3B).

To evaluate muscle force production, in situ force analysis was performed on the TA muscle. Male *Sgcg-*MRL TA muscle produced twice the maximum tetanic force compared with male *Sgcg*-D2 TA muscle (Figure 2E). This difference was not evident in female mice due to the high variability in muscle force measurements (Supplemental Figure 4A). The physiological CSA (PCSA) of TA muscles was larger for MRL mice compared with D2 mice for both males and females, consistent with the larger size of MRL compared with D2 mice (Figure 2F and Supplemental Figure 4B). In both male and female mice, specific force was similar between the *Sgcg*-MRL and *Sgcg*-D2 muscles, consistent with MRL muscles generating strength proportional to their larger size (Figure 2G and Supplemental Figure 4C).

### Upregulation of regenerative pathways in Sgcg-MRL muscles and increased inflammatory gene expression in Sgcg-D2 muscles.

**Figure 3:**
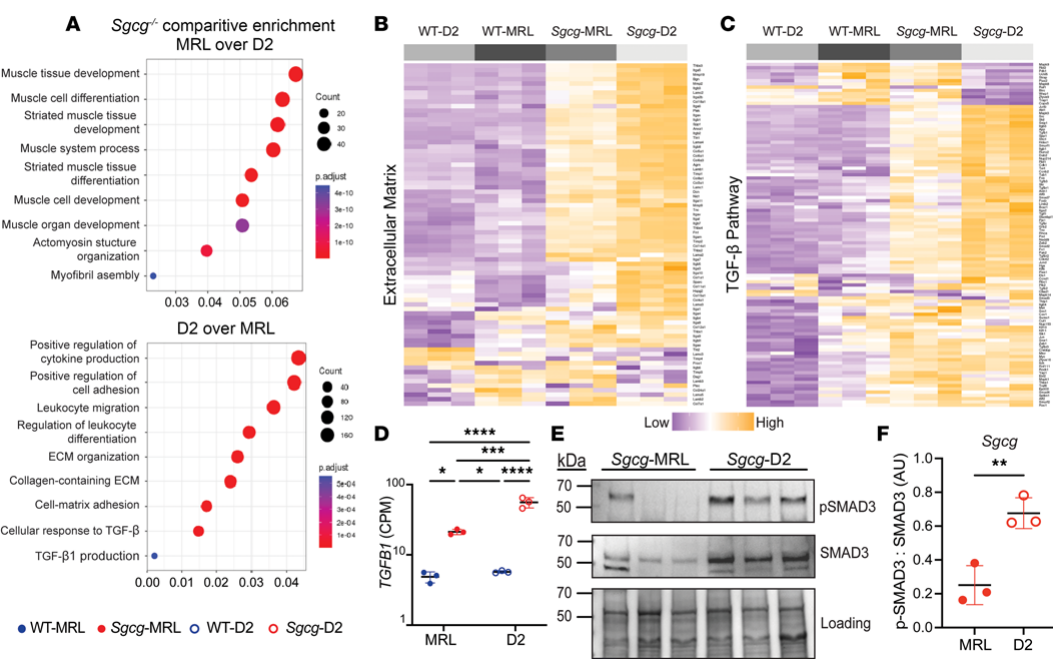
TGF-β gene expression and signaling were downregulated in *Sgcg*-MRL skeletal muscle compared with *Sgcg*-D2 muscle. RNA sequencing was performed on quadriceps muscles from 16-week-old female mice in biological triplicate. Gene expression was normalized to counts per million (CPM). (**A**) Comparison of *Sgcg*-MRL versus *Sgcg*-D2 transcriptomic profiles showed muscle development and differentiation genes to be highly enriched the *Sgcg*-MRL background (top). In contrast, immune response, extracellular matrix, and TGF-β pathways were highly enriched in the *Sgcg*-D2 cohort. (**B**) Clustered heatmap shows reduction of extracellular matrix genes in *Sgcg*-MRL muscle. (**C**) Clustered heatmap shows reduction in TGF-β pathway genes in *Sgcg*-MRL muscle. (**D**) Comparative log-scale analysis showed substantially higher average expression of *Tgfb1* in the *Sgcg*-D2 cohort (WT-MRL 4.86, *Sgcg*-MRL 21.3, WT-D2 5.73, and *Sgcg*-D2 55.9 CPM). (**E**) Immunoblotting of quadriceps muscles from *Sgcg*-D2 and *Sgcg*-MRL mice showed reduced phosphorylated SMAD3 (p-SMAD3) and total SMAD3 in *Sgcg*-MRL. (**F**) The ratio of p-SMAD3 to total SMAD3 was quantified and indicated TGF-β signaling was decreased in the MRL background. Data are presented as mean ± SD. *P < 0.05; **P < 0.01; ***P < 0.001; ****P < 0.0001 by 2-way ANOVA (**D**) or 2-tailed Student’s *t* test (**F**).

Bulk RNA-seq was performed on quadriceps muscles harvested from 16-week-old WT-MRL, *Sgcg*-MRL, WT-D2, and *Sgcg*-D2 mice. Principal component analysis of the RNA-seq data set revealed divergent clustering, with effects of both genetic strain and the *Sgcg^–/–^* disease mutation (Supplemental Figure 5). Direct comparison of the *Sgcg*-MRL and *Sgcg*-D2 muscles revealed the MRL background resulted in a greater expression of genes associated with muscle development and differentiation, while *Sgcg-*D2 tissue expressed genes linked to inflammatory responses, ECM development, and TGF-β signaling (Figure 3A). Representative heatmaps demonstrate that gene expression associated with the ECM and the TGF-β signaling pathway was upregulated in the D2 model (Figure 3, B and C). The full, clustered list of these genes is presented in Supplemental Figures 6 and 7. *Tgfb1* was upregulated in *Sgcg*-D2 and downregulated in *Sgcg*-MRL (Figure 3D). TGF-β signaling was evaluated in quadriceps muscle by immunoblot analysis and showed excess phosphorylated SMAD3 (p-SMAD3) in *Sgcg*-D2 muscle compared with *Sgcg*-MRL (Figure 3, E and F). Correspondingly, immunoblot analysis of the gastrocnemius/soleus muscles also demonstrated reduced p-SMAD3 in the *Sgcg*-MRL strain relative to *Sgcg*-D2 (Supplemental Figure 8).

### The MRL strain has a regenerative ECM.

**Figure 4:**
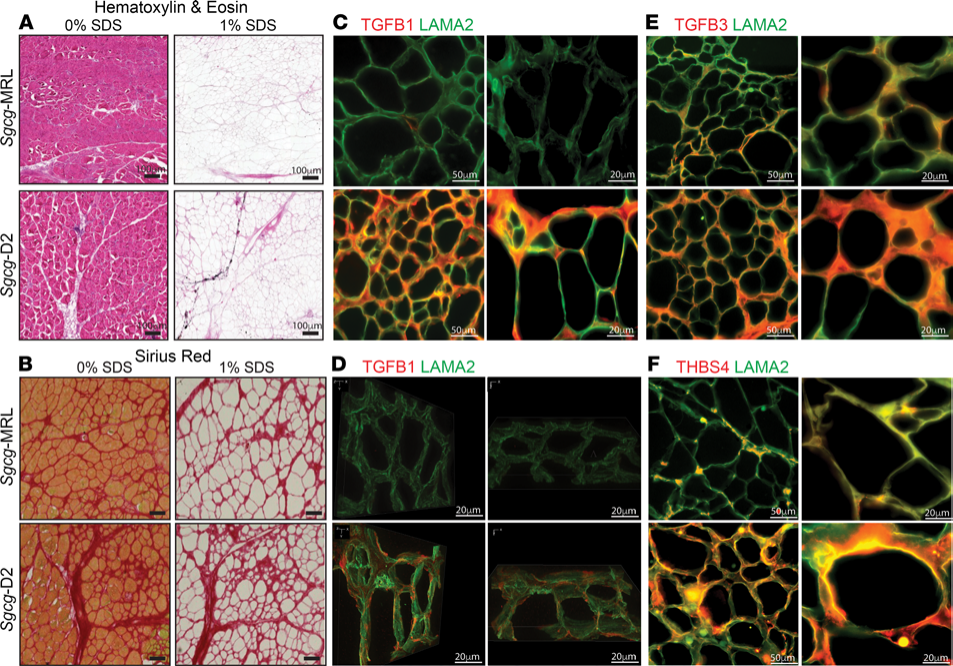
Reduction in TGF-β proteins in decellularized matrices from *Sgcg*-MRL muscle compared with *Sgcg*-D2 muscle. Decellularized extracellular (dECM) myoscaffolds were generated from *Sgcg*-MRL and *Sgcg*-D2 quadriceps muscles. Myoscaffold sections were stained with (**A**) H&E and (**B**) Sirius Red, demonstrating removal of cellular content following detergent treatment (1% SDS) with maintenance of matrix components. Control samples were incubated in PBS only (0% SDS). (**C**) Representative IFM images of dECM myoscaffolds costained with antibodies to detect TGF-β1 (red) and LAMA2 (green). Extracellular TGF-β1 protein expression was dramatically reduced in the MRL background. (**D**) *Z*-stack images of TGF-β1 (red) and LAMA2 (green) demonstrate excess TGF-β1 distributed throughout the matrix in the dystrophic D2 model. Original magnification, ×100. (**E**) Representative IFM images of dECM sections, costained with antibodies to detect TGF-β3 (red) and LAMA2 (green), showing reduction in TGF-β3 in *Sgcg*-MRL myoscaffolds. (**F**) dECM myoscaffolds from *Sgcg*-MRL (top) and *Sgcg*-D2 (bottom) showed reduced thrombospondin-4 (THBS4, red) and LAMA2 (green) in the *Sgcg*-MRL muscle. Scale bars: 100 μm (**A**), 50 μm (left images in **C**, **E**, and **F**), and 20 μm (**B**, **D**, and right images in **C**, **E**, and **F**).

The molecular composition of the ECM was investigated in decellularized ECM (dECM) prepared from *Sgcg*-MRL and *Sgcg*-D2 quadriceps muscles. This on-slide decellularization protocol was optimized to generate myoscaffolds following previously described methods (31, 32). *Sgcg*-MRL and *Sgcg*-D2 quadriceps sections were incubated in 1% SDS and subsequently stained with hematoxylin and eosin (H&E) and Sirius Red to confirm removal of cellular material and retention of an intact ECM (Figure 4, A and B, respectively). Representative images of dECMs confirm removal of intracellular components compared with control sections treated with 0% SDS, which retained the cellular components and validated the effectiveness of this decellularization method. *Sgcg*-MRL myoscaffolds displayed reduced collagen, evidenced through Sirius Red staining, compared with *Sgcg*-D2 myoscaffolds, consistent with the antifibrotic role of the MRL background in *Sgcg*-MRL muscle.

Representative images of dECM myoscaffolds costained with anti–TGF-β1 and anti-LAMA2 exhibited reduced TGF-β1 staining in the dECM of the *Sgcg*-MRL model (Figure 4C). Further visualization through 3D *Z*-stack imaging demonstrated minimal TGF-β1 in *Sgcg*-MRL scaffolds, while TGF-β1 was abundant throughout the matrix of *Sgcg*-D2 mice (Figure 4D). Similarly, anti–TGF-β3 and anti-LAMA2 costaining of the myoscaffolds revealed that TGF-β3 was also reduced by the MRL background (Figure 4E). Thrombospondin-4 (THBS4), a multifunctional glycoprotein that modifies muscular dystrophy outcome, was also markedly downregulated in the *Sgcg*-MRL myoscaffolds but was abundant in the *Sgcg*-D2 dECM (Figure 4F). Similarly, dECM from gastrocnemius/soleus muscles showed a marked reduction in TGF-β1, TGF-β3, and THBS4 in *Sgcg*-MRL muscle compared with *Sgcg*-D2 muscle (Supplemental Figure 9). Together, these results indicate a dramatic decrease in TGF-β signaling induced by the MRL background, which in turn, likely promotes the proregenerative nature of the MRL background.

### Sgcg-MRL dECM myoscaffold enhanced differentiation in seeded C2C12 myoblasts.

**Figure 5:**
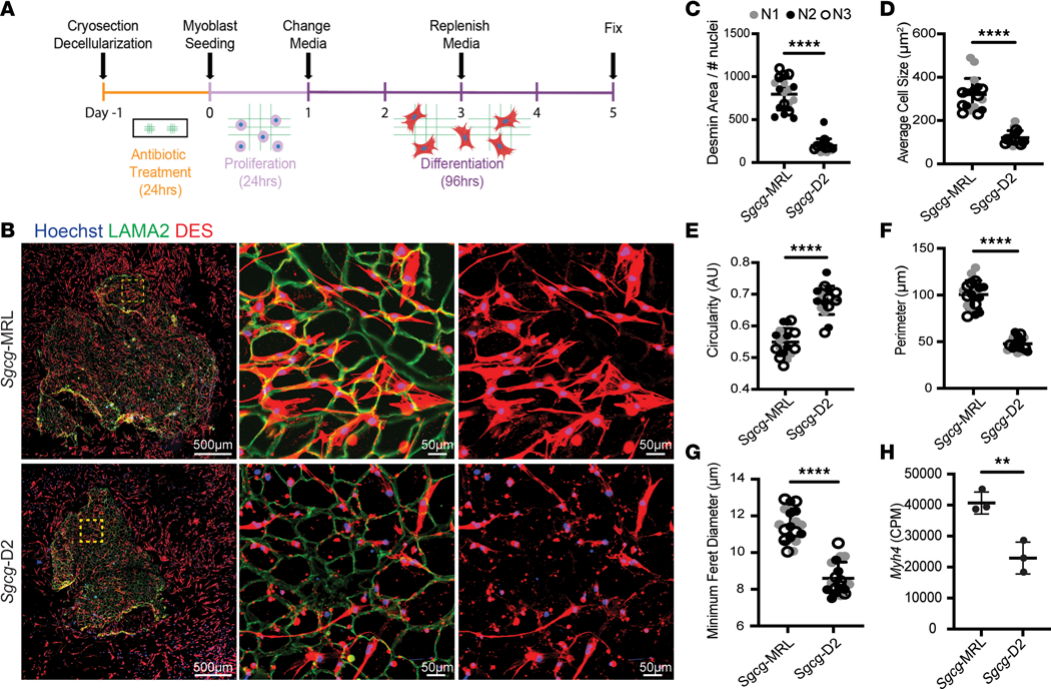
Decellularized myoscaffolds from *Sgcg*-MRL muscle promote migration and early differentiation of C2C12 myoblasts. (**A**) C2C12 myoblasts were seeded at low density and proliferated on dECM myoscaffolds from *Sgcg*-MRL (top) and *Sgcg*-D2 (bottom) for 24 hours. Then, growth media were replaced with low-serum differentiation media for 96 hours. Slides were fixed for imaging. (**B**) Imaging of dECM myoscaffolds seeded with C2C12 myoblasts. The left column (scale bars: 500 μm) represents entire myoscaffolds with seeded myoblasts costained for LAMA2 (green), desmin (DES, red), and with Hoechst (blue) to stain nuclei. The yellow region of interest is magnified in the middle and right columns (scale bars: 50 μm). DES (red) staining in the right column shows myoblast morphology. (**C**–**H**) Three independent biological replicates of the *Sgcg*-MRL and *Sgcg*-D2 dECMs were analyzed (labeled as N1 = gray, N2 = black, N3 = white). For each sample, 6 images of myoblasts were captured at ×20 magnification. (**C**) Area of desmin positivity relative to the number of nuclei in the field (*Sgcg*-MRL 797.0 and *Sgcg*-D2 197.4 mm^2^). (**D**) Average cell size (*Sgcg*-MRL 319.7 and *Sgcg*-D2 120.8 mm^2^). (**E**) Circularity (*Sgcg*-MRL.549 and *Sgcg*-D2.681 AU). (**F**) Myoblast perimeter (*Sgcg*-MRL 100.5 and *Sgcg*-D2 47.8 mm). (**G**) Minimum Feret diameter (*Sgcg*-MRL 11.5 and *Sgcg*-D2 8.6 mm). Quadriceps were isolated from 3 biological replicates of the *Sgcg*-MRL and *Sgcg*-D2 cohorts. (**H**) RNA sequencing was performed and *Myh4* gene expression was found to be upregulated in the MRL strain (*Sgcg*-MRL 40,692 and *Sgcg*-D2 22,915 CPM). Data are presented as mean ± SD. **P < 0.01, ****P < 0.0001 by 2-tailed Student’s *t* test.

The extracellular environment of skeletal muscle provides an essential substrate to support regeneration and formation of myotubes after injury (33, 34). Given the differential protein deposition in dECMs from *Sgcg*-MRL and *Sgcg*-D2 muscles, we investigated how these matrices altered myoblast morphology and differentiation potential. The fusion of mononucleated myoblasts into multinucleate myotubes relies on many factors, including myoblast density, cell motility, fusogenic capacity, and signaling cues present in the external environment (35, 36). We used C2C12 myoblasts across all experiments to maintain a consistent cell source and allow for direct comparison. We first evaluated C2C12 myoblasts seeded sparsely onto dECMs to study myoblast migration properties, since at low density, myoblasts will have restricted contact with neighboring cells, limiting formation of multinucleate myotubes upon serum withdrawal. Following a 5-day differentiation protocol, the slides were fixed and stained with antibodies against desmin and LAMA2 (Figure 5, A and B). Desmin is a muscle-specific intermediate filament protein that is one of the earliest markers of myogenic commitment (37, 38). Lack of desmin impairs muscle regeneration and differentiation (39). Desmin-positive cells on *Sgcg*-MRL myoscaffolds were larger and covered more area compared with myoblasts similarly seeded on *Sgcg*-D2 myoscaffolds. On *Sgcg*-D2 scaffolds, desmin-positive cells were small, with little cellular area, consistent with poor differentiation (Figure 5B). Desmin expression and cell morphology were quantified using ImageJ software from 6 representative images captured from 3 *Sgcg*-MRL and *Sgcg*-D2 biological replicates. When standardized to the number of nuclei, the percentage area of desmin fluorescence was significantly increased in myoblasts seeded on the *Sgcg*-MRL myoscaffolds compared with those seeded on the *Sgcg*-D2 myoscaffolds (Figure 5C). Myoscaffold strain background had a marked influence on the average cell size, circularity, and perimeter (Figure 5, D–F, respectively), where MRL-seeded myoblasts exhibited an increase in cell size and had a less circular morphology. Similarly, myoblasts seeded on MRL scaffolds exhibited a significant increase in minimum Feret diameter (Figure 5G). These measures indicate that the dECM myoscaffolds of the MRL strain promoted greater growth and development of myoblasts.

**Figure 6:**
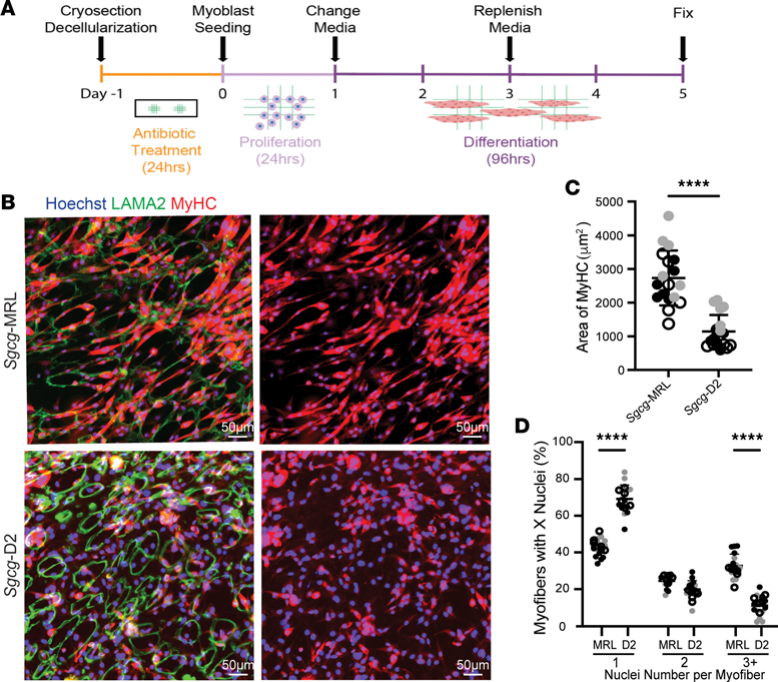
Decellularized myoscaffolds from *Sgcg*-MRL muscle promote differentiation of C2C12 myoblasts into myotubes. Experiments similar to those in Figure 5 were conducted, except that myoblasts were seeded at higher density to promote fusion to myotubes. (**A**) C2C12 myoblasts were seeded at high density onto myoscaffolds and differentiated. (**B**) Imaging of C2C12 myoblasts seeded onto *Sgcg*-MRL (top) and *Sgcg*-D2 (bottom) dECM myoscaffolds costained with antibodies against MyHC-2B (red) and LAMA2 (green), and with Hoechst (blue). Myoblasts were seeded onto 3 biological replicates per cohort and 6 images were analyzed. Original magnification, ×20. Scale bars: 50 μm. (**C**) The area of MyHC-2B was significantly greater on *Sgcg*-MRL myoscaffolds (*Sgcg*-MRL 2,736 and *Sgcg-*D2 1,143 μm^2^). (**D**) Singly nucleated myofibers were increased on *Sgcg*-D2 dECMs (69.2%) compared with *Sgcg*-MRL dECMs (42.8%). There was significantly more myofibers with 3 or more nuclei on *Sgcg*-MRL dECMs (33.7%) compared with *Sgcg*-D2 dECMs (11.1%), indicating enhanced myotube formation. Data are presented as mean ± SD. ****P < 0.0001 by 2-tailed Student’s *t* test (**C**) or 2-way ANOVA with Tukey’s multiple-comparison test (**D**).

*Myh4* encodes fast myosin heavy chain 2B (MyHC-2B) and is expressed once myoblasts fuse to form myotubes and in mature myofibers (40). The *Myh4* transcript level in whole quadriceps muscle was increased in the *Sgcg*-MRL compared with the *Sgcg*-D2 strain, consistent with enhanced muscle differentiation, especially myotube formation, in vivo (Figure 5H). To interrogate the ECM’s effect on myotube formation, C2C12 cells were seeded onto myoscaffolds at higher density to promote cell-cell contract and allowed to differentiate for 5 days, comparing *Sgcg*-MRL and *Sgcg*-D2 dECMs (Figure 6, A and B). Here, we evaluated MyHC-2B as a marker of myotube formation. The average MyHC-2B–positive cell size was significantly increased in muscle cells residing on the *Sgcg-MRL* myoscaffolds compared with cells on *Sgcg-*D2 myoscaffolds (Figure 6C). Additionally, the percentage of total MyHC-2B area per field was significantly increased in the *Sgcg*-MRL strain compared with the *Sgcg*-D2 strain (Figure 6D). To measure the impact of the matrix strain on myoblast fusion potential, the number of nuclei per MyHC-2B–positive myotubes was quantified. *Sgcg*-MRL myoscaffolds supported a significantly increased number of multinucleate MyHC-2B–positive myotubes compared with *Sgcg*-D2 myoscaffolds (Figure 6B). Together, these data show a strain-dependent effect of the ECM that impacts myoblast differentiation and fusion capacity.

### The MRL background has increased circulating growth factors.

**Figure 7:**
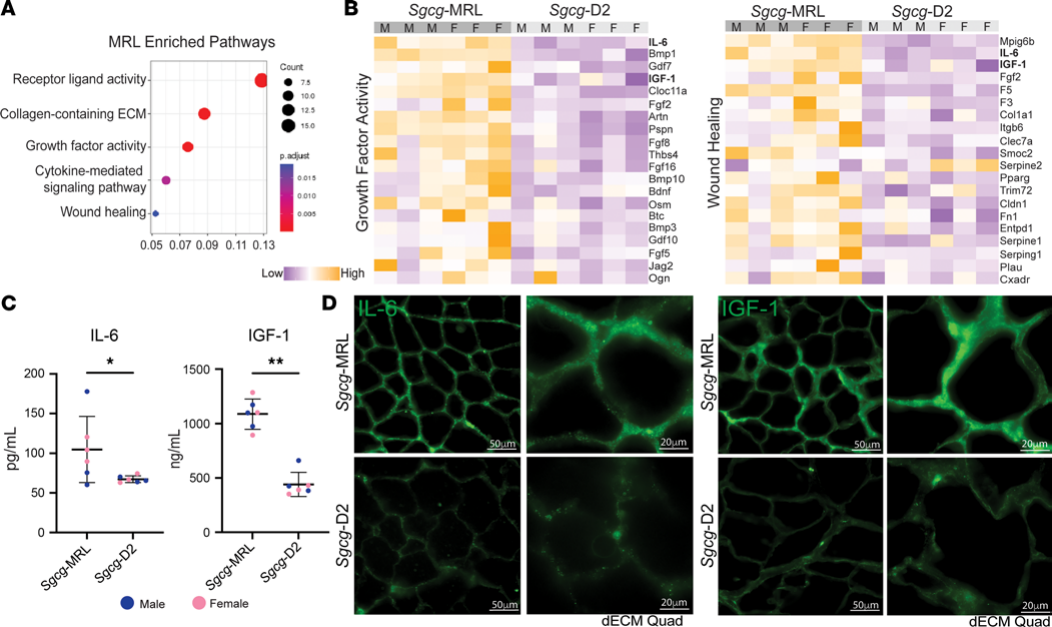
IGF-1 and IL-6 were upregulated in *Sgcg*-MRL serum. Serum was collected from *Sgcg*-MRL and *Sgcg*-D2 cohorts at 20 weeks of age and evaluated using the SomaScan aptamer assay. (**A**) Pathway enrichment analysis showed receptor ligand, collagen-containing ECM, and growth factor activity highly enriched in *Sgcg*-MRL serum. (**B**) Heatmaps of proteins in the growth factor activity (left) and wound healing (right) pathways upregulated in *Sgcg*-MRL mice. IL-6 and IGF-1 (bold) were among the most differentially expressed proteins in both pathways. (**C**) IL-6 and IGF-1 upregulation was verified using ELISA analysis. Data are presented as mean ± SD. *P < 0.05, **P < 0.01 by Kolmogorov-Smirnov test. (**D**) Matrix deposition of IL-6 and IGF-1 increased in decellularized myoscaffolds from *Sgcg*-MRL but not *Sgcg*-D2 muscles. Scale bars: 50 μm (left columns) and 20 μm (right columns).

The capacity of skeletal muscle to repair and regenerate after injury is directly influenced by circulating cytokines and growth factors (41, 42). We used the SomaScan aptamer assay to assess the proteomic profile of serum collected from the *Sgcg*-MRL and *Sgcg*-D2 cohorts at 20 weeks of age. Comparative enrichment analysis between the models found the MRL serum to harbor increased expression of proteins associated with growth factor activity and wound healing (Figure 7A). Heatmaps depicting differentially expressed proteins (FDR *P* value < 0.1) associated with growth factor activity (top) and wound healing (bottom) indicate upregulation in the MRL strain, where the samples are ordered by sex and significance. Insulin-like growth factor 1 (IGF-1) and IL-6 were among the most differentially expressed proteins in both pathways (Figure 7B). This analysis also identified increased FGF2 expression in *Sgcg*-MRL, but we did not explore this protein further because of the high variability between samples (Supplemental Figure 10). Enzyme-linked immunosorbent assays (ELISAs) were conducted in the same *Sgcg* serum samples for IL-6 and IGF-1 (Figure 7C) and confirmed upregulation in *Sgcg*-MRL compared with *Sgcg*-D2. To evaluate whether these shifts were evident in WT backgrounds, we conducted serum ELISA for IL-6 or IGF-1 from WT-MRL and WT-D2 mice. IL-6 levels were similar between WT-MRL and WT-D2, indicating a specific upregulation of serum IL-6 in *Sgcg*-MRL mice (Supplemental Figure 11A and Figure 7). Serum IGF-1 was higher in WT-MRL than WT-D2, similar to *Sgcg*-MRL and *Sgcg*-D2 mice (Supplemental Figure 11B and Figure 7). Myoscaffolds from *Sgcg*-MRL muscles had marked accumulation of both IL-6 and IGF-1 compared with *Sgcg*-D2 myoscaffolds (Figure 7D). However, dECM from WT muscles did not demonstrate enhanced accumulation of either IL-6 or IGF-1 in the matrix (Supplemental Figure 11, C and D). Therefore, the deposition of IL-6 and IGF-1 myokines in the ECM is specific to *Sgcg*-MRL mice, as neither were observed in ECM from WT-MRL nor WT-D2 muscles. These data support a mechanism where serum myokines in MRL mice are recruited to the dystrophic matrix to support enhanced regeneration.

## Discussion

### The MRL background promotes sustained muscle regeneration in muscular dystrophy.

The enhanced wound healing properties of the MRL strain have been described after many different forms of acute injury like ear-hole punching or acute injury of cartilage or skin (15, 17). Many of these studies evaluating the wound healing properties of the MRL background were conducted on younger animals, since with age the MRL model is susceptible to autoimmune disorders (43, 44). We focused our study on the first 20 weeks of age, finding evidence for a sustained contribution of the MRL background to improve the severe fibrosis characteristic of the dystrophic *Sgcg-*null allele. In mice and humans with muscular dystrophy, the diaphragm and respiratory muscles are highly impaired (45, 46), and so it is notable that the MRL background was able to correct functional deficits in the dystrophic mouse model. The protective qualities of the MRL background were not restricted to just the diaphragm muscle since the other studied muscles also showed functional and histological improvement, including marked reduction of fibrosis and enhanced features of muscle regeneration. Muscular dystrophy preferentially degrades fast twitch myofibers (type IIB), causing a fiber type shift from fast to slow twitch (type I) (47, 48). The MRL background protected against this fiber type shift, where a higher proportion of MyHC-positive fibers (type IIB) were present, and MYH7-positive fibers (type I) were almost completely absent. It is possible over even longer periods of time that the MRL background may begin to uncover some of the autoimmune features in this background.

### Sex influences body mass composition in the MRL strain.

MRL mice are larger than many other mouse strains, nearly twice the size of other mice, and along with this, the MRL strain carries a distinct metabolic profile (18, 49, 50). Embryonic features of the metabolism are retained longer in MRL mice, and these features have also been associated with decreased reactive oxygen species (ROS), and consequent stem cell activation and tissue regeneration (49). MRL mitochondria contain 2 naturally encoded variants, which have been found to decrease severity of muscle disease (51). It has been shown that MRL mice adapted to a high-fat diet by metabolically shifting from carbohydrate metabolism toward β-oxidation. In this setting of high-fat diet, MRL mice still experienced weight gain, but this metabolic shift protected against cardiac hypertrophy, hyperglycemia, and insulin resistance (50). Although we observed no difference in overall body mass between male and female WT-MRL mice, the body mass composition varied considerably, with females gaining significantly more fat mass than their male counterparts. This sexually dimorphic pattern was only seen in WT-MRL and was not apparent in *Sgcg*-MRL, WT-D2, and *Sgcg*-D2 mice.

### Suppression of TGF-β signaling in the MRL background.

The TGF-β proteins (β1, β2, β3) promote fibrosis in many settings, including muscular dystrophy (23, 52, 53). In part, TGF-β–induced fibrosis is dictated by genetic modifiers like *LTBP4* and *SPP1* (25, 54). The MRL background carries the protective *Ltbp4* polymorphism that suppresses active TGF-β in dystrophic muscle. Polymorphisms of *LTBP4* in the D2 background are associated with increased TGF-β signaling through SMAD2/3 phosphorylation (54, 55). The activation of SMAD2/3 upregulates expression of ECM-associated genes (56), and the D2 strain carries an allele of *Ltbp4* that enhances active TGF-β, contributing to the exacerbation of muscular dystrophy by the D2 background (12). We demonstrate that TGF-β signaling is suppressed, with SMAD2/3 phosphorylation, in the MRL background. This reduction in TGF-β signaling corresponded to enhanced cell growth features in myoblasts seeded onto the MRL dECM. These findings are consistent with other injury settings, including cartilage (57), where downregulation of TGF-β signaling strongly correlated with improved cartilage repair.

Decellularized ECM scaffolds are useful platforms to study matrisomal protein composition and tissue regeneration (58–60). We employed the decellularization methods developed by Stearns-Reider et al. (27) and found that the highly fibrotic *Sgcg*-D2 muscles were characterized by excess TGF-β1 deposited throughout the ECM. In contrast, the MRL background had little to no TGF-β1 in its ECM. Similarly, expression of ECM proteins TGF-β3 and THBS4 was notably diminished in the MRL background. THBS4 is a key protein in the assembly of the ECM after injury and promotes tendon strength and repair (61). Analysis of human muscle biopsies from patients with muscular dystrophy found enriched THBS4 expression, consistent with dystrophic mouse models (62, 63). The reduction in THBS4 and TGF-β proteins in MRL ECM corresponds to the decreased collagen accumulation and is consistent with markedly delayed progression of the muscular dystrophy process.

### The MRL harbors a regenerative ECM that promotes muscle growth.

Components of the ECM can provide instructive cues during muscle regeneration (33, 64). Muscle regeneration involves a multifaceted, coordinated response that can be interrupted by inflammatory factors (21), including those components present in dystrophic muscle (62, 65). We found that the dECM derived from the dystrophic MRL background promoted an increase in myoblast size and muscle differentiation capacity, indicating cell-extrinsic healing properties of the MRL background. We found the serum of *Sgcg*-MRL mice to be enriched with proteins associated with growth factor activity and wound healing. While excess TGF-β acts as a potent inhibitor of myoblast differentiation, IGFs stimulate proliferation and regeneration (41). Media supplementation with IGF-1 is known to stimulate C2C12 myoblasts to upregulate expression of *Myh4* and form myotubes (66). Similarly, our studies showed that MRL ECM with increased IGF-1 matrix deposition stimulated seeded myoblasts to upregulate *Myh4* gene expression and enhance MyHC production. Correspondingly, these matrices facilitate myoblast maturation into myotubes. The increased circulation of IGF-1 and IL-6 are positioned to mediate the proregenerative qualities observed in the MRL ECM through enhanced deposition into the muscle ECM (67–69). IGF-1 is a circulating growth factor that promotes anabolic pathways in skeletal muscle and prevents age-related sarcopenia (67). Overexpression of IGF-1 reduces muscle pathology and stabilizes the sarcolemma in the *mdx* model (70). IL-6 is a secreted glycoprotein that also serves as a myokine, where it is necessary for muscle regeneration (69, 71, 72). Upregulation of IL-6 has been observed in MRL serum, where it has been thought to contribute to autoimmune conditions (73). However, in this setting, the increase in IL-6 is also present as ECM deposition of this myokine, where it can directly contribute to muscle regeneration and the protective, proregenerative microenvironment observed in the *Sgcg*-MRL model. Although we focused on IL-6 and IGF-1, other growth factors may also contribute. This study demonstrates the fundamental influence genetic background has on dystrophic progression and highlights the importance of the extracellular microenvironment in muscle regeneration and repair.

## Methods

### Animals.

WT-D2 mice were from the DBA/2J strain (Jackson Laboratory, stock 000671) and (WT-MRL) were from the MRL/Mpj strain (Jackson Laboratory, stock 000486). The *Sgcg*-null mice were previously generated by deleting exon 2 and then breeding more than 10 generations in the B6 background (8). Mice were backcrossed over 10 generations with the DBA/2J (*Sgcg-D2*) and MRL (*Sgcg-MRL*) strains. Male and female mice were used for all experiments, unless otherwise noted. Mice were bred and housed in a specific pathogen–free facility on a 12-hour light/dark cycle and fed ad libitum. Where possible, physiological analyses were performed blinded to genotype and background strain, although the size/appearance differences between MRL and D2 made blinding to this aspect impossible. Cohorts were randomized to ensure even sex distribution across cohorts and approximate age matching.

### Histology.

Quadriceps muscle was harvested and fixed in 10% formalin. H&E (12013B and 1070C, Newcomer Supply), Masson’s trichrome (HT15, Newcomer Supply), and Sirius Red (24901250, Polysciences, Inc) staining was performed per manufacturers’ protocol. For all genotypes, images were acquired on the Keyence BZ-X810 microscope with the same exposure settings. Masson’s trichrome image assessment was performed on representative ×20 images from 3 male biological replicates using ImageJ (NIH), where the fibrotic content was standardized to full muscle area.

### Body weight and mass composition analysis.

Total body mass was determined at 10, 15, and 20 weeks. Fat mass, lean mass, and hydration ratio were determined at 20 weeks of age, using the noninvasive NMR method provided by EchoMRI. Each cohort consisted of 10 mice, 5 male and 5 female.

### Plethysmography.

Unanesthetized whole-body plethysmography (WBP) was performed at 10 weeks using a Data Sciences International Buxco Finepointe 4-site WBP as described previously (74, 75). Chambers were calibrated and then individual mice were placed inside the chamber for a 120-minute acclimation period prior to experimental recording. Data were acquired for 10 minutes while mice were in a resting phase. Studies were performed at room temperature. Data points were filtered to include breaths with a 0 Rejection Index (Rinx) and within a frequency range of 100–250 breaths per minute. Penh was normalized to body mass (g).

### Serum collection.

Serum was acquired and processed as described previously (76). Briefly, using a heparinized capillary tube (20-362-566, Thermo Fisher Scientific) blood was collected by means of retro-orbital puncture into a Microtainer Gold Top Serum Separator tube (365967, Becton Dickinson) and centrifuged at 8,000*g* for 10 minutes. Serum fractions were collected at 20 weeks of age and stored at −80°C.

### Serum-based ELISAs for circulating proteins.

Serum fractions from the *Sgcg*-MRL and *Sgcg*-D2 cohorts were collected in 3 male and 3 female mice at 20 weeks of age. IL-6 and IGF-1 serum levels were assessed using the Mouse IL-6 Quantikine ELISA Kit (M6000B, R&D Systems) and Mouse IGF-1 Quantikine ELISA kit (MG100, R&D Systems), respectively, according to the manufacturer’s instructions.

### CK measurement.

Following the manufacturer’s instructions, serum was analyzed in duplicate for each mouse using the EnzyChrom Creatine Kinase Assay (ECPK-100, BioAssay Systems). *Sgcg* serum was diluted 1:2 for assays, while WT serum was assayed undiluted. Results were acquired with the Synergy HTX multimode plate reader (BioTek).

### Serum aptamer profiling assay and analysis.

The SomaScan assay reports 7,322 aptamer-based proteomics results per sample in relative fluorescent units, which were read into R studio using the SomalogicsIO R package (https://www.r-project.org/). Linear mixed modeling was performed using the dream package in R. Data were imported and preprocessed, and a linear mixed model was fit to the data using the lme function. Model fit and estimated parameters were obtained using the summary function, and fixed and random effect estimates were extracted using the fixef and ranef functions, respectively. Model diagnostics, including checking the normality of the residuals, visualizing the residuals against the predicted values, and checking for heteroscedasticity and outliers, were performed using the qqmath, plot, and plot functions. Hypothesis tests were conducted using the anova function, and confidence intervals for the estimated parameters were computed using the confint function.

### In situ force and fatigue.

The TA was assayed using an Aurora Scientific 1300A Whole Animal System. Mice were anesthetized via isoflurane, and the initial muscle isolation was performed under a higher isoflurane concentration (1 L/min of 2.0% isoflurane in 100% O_2_). To prepare the TA for in situ force measurement, an incision was made in the skin from the anterior side of the left foot up toward the left knee. This allowed for access to both the distal TA tendon and the anterior compartment of the lower hindlimb. Once the fascia was removed, the distal TA tendon was cut, and the TA was isolated via blunt dissection. A braided silk suture (4-0 diameter) was tied around the distal tendon before the mouse was transferred to the experimental setup. The anesthesia was then reduced (1 L/min or 1.5% isoflurane in 100% O_2_) to minimize any deleterious effects induced by long-term exposure. It was noted during setup that MRL mice required a higher dose of isoflurane (2%–2.5% during isolation, 1.8%–2% during experimentation). This is likely due to the larger size of the MRL model. The free end of the tendon suture was then tied to the lever arm of a 5-N dual-action force transducer (305C-LR, Aurora Scientific). Slack length was removed, and 2 fine-needle electrodes were positioned on either side of the midline in the belly of the TA. An optimal stimulation amperage was determined via repeated twitch contractions at 0.5 Hz. A tetanic contraction was performed to remove slack from the system before the muscle’s optimal length (L_0_) was determined through the same process. Maximum tetanic forces (77) were normalized to the PCSA. The PCSA was determined through the following equation: PCSA (mm^2^) = *M* (mg) × cos(*θ*)/*ρ* (mg/mm^3^) × *L_f_* (mm), where *M* is the wet weight of the TA, *θ* is the pennation angle of the TA, *ρ* is the physiological density of muscle (1.056 mg/mm^3^), and *L_f_* is the fiber length of the TA. *L_f_* was converted from the optimal muscle length through the observed fiber length to muscle length ratio of 0.61. To determine the half-maximal frequency of the force-frequency relationship, the data were fitted to a sigmoidal curve (78). To determine percentage force loss as caused by the fatigue protocol, the final tetanic contraction was compared to the starting tetanic contraction.

### Fiber type analysis.

Fiber typing followed the methods described in Salamone et al. (79). Frozen muscle sections (10 μm) of the gastrocnemius muscle were fixed in ice-cold acetone for 5 minutes, rinsed in PBS, and then blocked in 1% bovine serum albumin (BSA) and 10% fetal bovine serum (FBS) in PBS for 1 hour. For fiber typing, sections were incubated with primary antibodies BA-F8 (1:10), SC-71 (1:30), and BF-F3 (1:10), all from the Developmental Studies Hybridoma Bank, overnight at 4°C. Sections were rinsed in PBS plus 1% BSA and then incubated with secondary antibodies Alexa Fluor 350 anti-IgG2b, Alexa Fluor 488 anti-IgG1, and Alexa Fluor 594 anti-IgM (A21140, A21121, and 1010111, respectively; Life Technologies) (all used at 1:500) for 1 hour. Following secondary incubation, sections were again rinsed in PBS plus 1% BSA. All primary and secondary antibodies were diluted in a 1% BSA/PBS solution. All steps were carried out at room temperature using room temperature reagents except where noted. All samples were fixed in ProLong Gold Antifade Mountant (P36930, Thermo Fisher Scientific) and imaged on a Keyence microscope. Type 2B, type 2A, and type 1 fibers were quantified and expressed as the percentage of total myofibers per muscle section.

### CSA analysis.

TA muscles were dissected and frozen in liquid nitrogen. Frozen muscle sections (10 μm) of TA muscle were fixed in 4% paraformaldehyde for 5 minutes, rinsed in PBS, and then blocked in 1% BSA and 10% FBS in PBS for 1 hour. Anti-LAMA2 was used at 1:100 (catalog L0663, Sigma-Aldrich). Secondary Alexa Fluor 488 goat anti-rat (catalog A11006, Invitrogen) was used at 1:2,500. Anti-LAMA2 sarcolemmal fluorescence was used to outline individual myofibers to assess individual myofiber CSA automatically using the MatLab program SMASH (80), as described in Salamone et al. (79). The entire TA was imaged for analysis using the Keyence microscope fitted with an ×10 objective using the Keyence tiling feature.

### RNA isolation, sequencing, and analysis.

RNA was isolated from whole quadriceps muscle in the WT and *Sgcg^–/–^* models of the MRL and D2 strains. This was performed in biological triplicate, where 3 female mice 16 weeks of age were used per cohort. TRIzol (15596018, Life Technologies) was used to isolate the RNA, which was then filtered with an Aurum Total RNA Mini-kit (7326820, Bio-Rad Laboratories) and suspended in 30 μL of RNase-free water. The RNA samples were indexed and pooled for 100-bp single-end sequencing. The Illumina RNA Sample Prep Kit version 2.0 generated libraries from these quadriceps and aligned to mm10, following the methods of Mikovic et al. (81). Quantitation and analysis of transcripts was performed using EdgeR (https://www.r-project.org/) and counts per million (CPM) were used to calculate differential expression. Heatmaps were generated from *z* scores calculated from gene CPM values.

### Protein isolation.

Quadriceps muscles were harvested and flash frozen in liquid nitrogen. Tissues were ground with a mortar and pestle and lysed in whole tissue lysis buffer (50 mM HEPES pH 7.5, 150 mM NaCl, 2 mM EDTA, 10 mM NaF, 10 mM Na-pyrophosphate, 10% glycerol, 1% Triton X-100, 1 mM phenylmethylsulfonyl fluoride, 1× cOmplete Mini protease inhibitor cocktail [11836170001, Roche], 1× PhosSTOP [04906837001, Roche]). The protein concentration was measured with the Quick Start Bradford 1× Dye Reagent (5000205, Bio-Rad Laboratories).

### Immunoblotting.

Protein lysate (15 μg) diluted in 4× Laemmli buffer (1610747, Bio-Rad Laboratories) was resolved in 4%–15% Mini-PROTEAN TGX Precast Protein Gels (4561086, Bio-Rad Laboratories) and transferred to Immun-Blot PVDF Membranes for Protein Blotting (1620177, Bio-Rad Laboratories). Membranes were blocked using StartingBlock T20 (TBS) Blocking Buffer (37543, Thermo Scientific Scientific). Primary antibodies anti–p-SMAD3 (catalog ab52903, Abcam) and anti-SMAD3 (catalog ab40854, Abcam) were used at 1:1000. Secondary antibodies conjugated with horseradish peroxidase were used at 1:2,500 (catalog 111035003, Jackson ImmunoResearch Laboratories). ECL Substrate (1705061, Bio-Rad Laboratories) was briefly applied to the membranes and visualized using an iBright 1500 Imaging System (Invitrogen). Pierce Reversible Protein Stain Kit for PVDF Membranes (including MemCode; 24585, Thermo Fisher Scientific) was used to ensure equal loading. Band intensity was quantified using the gel tool in ImageJ.

### Decellularization.

Decellularization was performed as described in Stearns-Reider et al. (31), with previously described modifications (31, 32). Briefly, flash-frozen quadriceps muscle was cryosectioned at 25 μm (CM1950, Leica) and mounted on charged Superfrost Plus Microscope Slides (1255015, Thermo Fisher Scientific). Slides were stored at –80°C until decellularization. To decellularize the tissue, slides were thawed at room temperature for 1 hour and then placed in 15 mL of 1% UltraPure SDS solution (15553035, Invitrogen) for 5–10 minutes with agitation (40 rpm). Slides were rinsed in PBS with calcium and magnesium (21030CV, Corning) for 45 minutes followed by a 30-minute rinse in UltraPure Distilled H_2_O (10977015, Invitrogen) and then an additional 45-minute wash in PBS. Scaffolds were then fixed for immunostaining or used for live cell studies.

### Myoscaffold immunostaining and immunofluorescent imaging.

Decellularized muscle cryosections (dECMs) were fixed in 4% paraformaldehyde, rinsed, and blocked with a 1% BSA/10% FBS blocking buffer at room temperature for 1 hour. Scaffolds were incubated in primary antibodies overnight at 4°C at 1:100: anti-LAMA2 (α-2 chain) (catalog L0663, MilliporeSigma), anti–TGF-β1 (catalog 218981AP, Proteintech and catalog MA515065, Invitrogen), anti–mIL-6 (catalog AF-406-NA, R&D Systems), anti–mIGF-1 (catalog AF791, R&D Systems), anti-THBS4 (catalog AF2390, R&D Systems), and anti–TGF-β3 (catalog ab15537, Abcam). Secondary antibodies were used at 1:2,500 for 1 hour at room temperature: Alexa Fluor 488 goat anti-rat (catalog A11006, Invitrogen), Alexa Fluor 594 goat anti-rabbit (catalog A11012, Invitrogen), and Alexa Fluor 594 donkey anti-goat (catalog A11058, Invitrogen). The dECMs were mounted with ProLong Gold Antifade Reagent (P36934, Invitrogen). Images were acquired on a Keyence BZ-X810 microscope with identical exposure settings across genotypes.

### C2C12 differentiation and quantification on dECM.

Quadriceps muscles were cryosectioned and decellularized using the dECM method stated above. To reduce variability, scaffolds from all genotypes were generated and seeded in a single batch. Chambers from MatTek Two Well Cell Culture slides (CCS-2, MatTek) were applied to the slides containing the dECM myoscaffolds. The myoscaffolds were incubated for 24 hours in growth media (DMEM; 11995-073, Thermo Fisher Scientific) with 10% FBS and 1% penicillin/streptomycin (15070-063, Thermo FIsher Scientific). C2C12 myoblasts were seeded onto the myoscaffolds at a density of 100,000 cells in 3 mL of growth media for the myoblast study and 300,000 cells in 3 mL for the fusion study. After 24 hours, growth media were removed and replaced with 3 mL of differentiation media (DMEM with 2% horse serum [26050088, Thermo Fisher Scientific] and 1% penicillin/streptomycin). Seeded scaffolds were incubated in differentiation media for 96 hours and subsequently fixed in 4% paraformaldehyde. Scaffolds were blocked for 1 hour in 1% BSA/10% FBS and then costained with anti-desmin (1:100; catalog 16520-1-AP, Proteintech), anti–MyHC-2B (1:1000; catalog PA5-50065, Thermo Fisher Scientific), and anti-LAMA2 (1:100; catalog L0663, Sigma-Aldrich). Alexa Fluor 488 goat anti-rat (catalog A-11006, Invitrogen) and Alexa Fluor 594 goat anti-rabbit (catalog A-11012, Invitrogen) secondary antibodies were used at 1:2,500. Scaffolds were imaged identically on the Keyence BX-810 microscope using ×10 and ×20 objectives. Eighteen images per strain were acquired and quantified (obtained from 3 or more unique animals per strain, 2 scaffolds per animal, 3 or more images per scaffold). Desmin area, MyHC-2B area, cell morphological parameters, and nuclei count were quantified for each image using ImageJ.

### Statistics.

Statistical analyses were performed with Prism (GraphPad). When comparing 2 groups, 2-tailed Student’s *t* test with Welch’s correction (unequal variances) was used, unless otherwise noted. When comparing 3 or more groups of data for only 1 variable, 1-way ANOVA with Tukey’s multiple-comparison test was used. When comparing data groups for more than 1 related variable, 2-way ANOVA was performed. A *P* value less than or equal to 0.05 was considered significant. Error bars represent ±standard deviation (±SD).

### Study approval.

All procedures using mice followed the NIH *Guide for the Care and Use of Laboratory Animals* (National Academies Press, 2011) and were approved by Northwestern University’s Institutional Animal Care and Use Committee.

### Data availability.

RNA-seq data are deposited in NCBI’s Gene Expression Omnibus data repository (GEO GSE235368). All other data are available in the supplemental Supporting Data Values file.

## Author contributions

JGO, AML, JK, GL, and ARD performed experiments. JGO, ARD, ABW, and AHV evaluated the RNA-seq and aptamer analysis. JGO and FWL performed muscle mechanics studies and analysis. PGTP carried out muscle histology. MH performed mouse husbandry and plethysmography. RHC and MJS provided critical input in experimental methods. JGO, ARD, and EMM conceived the studies, analyzed the data, and wrote the manuscript.

## Supplementary Material

Supplemental data

Supplemental data set 1

Supporting data values

## Version 1 01/04/2024

In-Press Preview

## Version 2 02/08/2024

Electronic publication
