# Supplementary material for: The super-healing MRL strain promotes muscle growth in muscular dystrophy through a regenerative extracellular matrix
Source: JCI Insight. 2024 Jan 4;9(3):e173246. doi: 10.1172/jci.insight.173246 (PMC11143963; doi:10.1172/jci.insight.173246)
Supplement: Supplemental data [file jciinsight-9-173246-s063.pdf]

SUPPLEMENTAL INFORMATION
The super-healing MRL strain promotes muscle growth in muscular dystrophy through a regenerative extracellular matrix
O'BRIEN ET AL

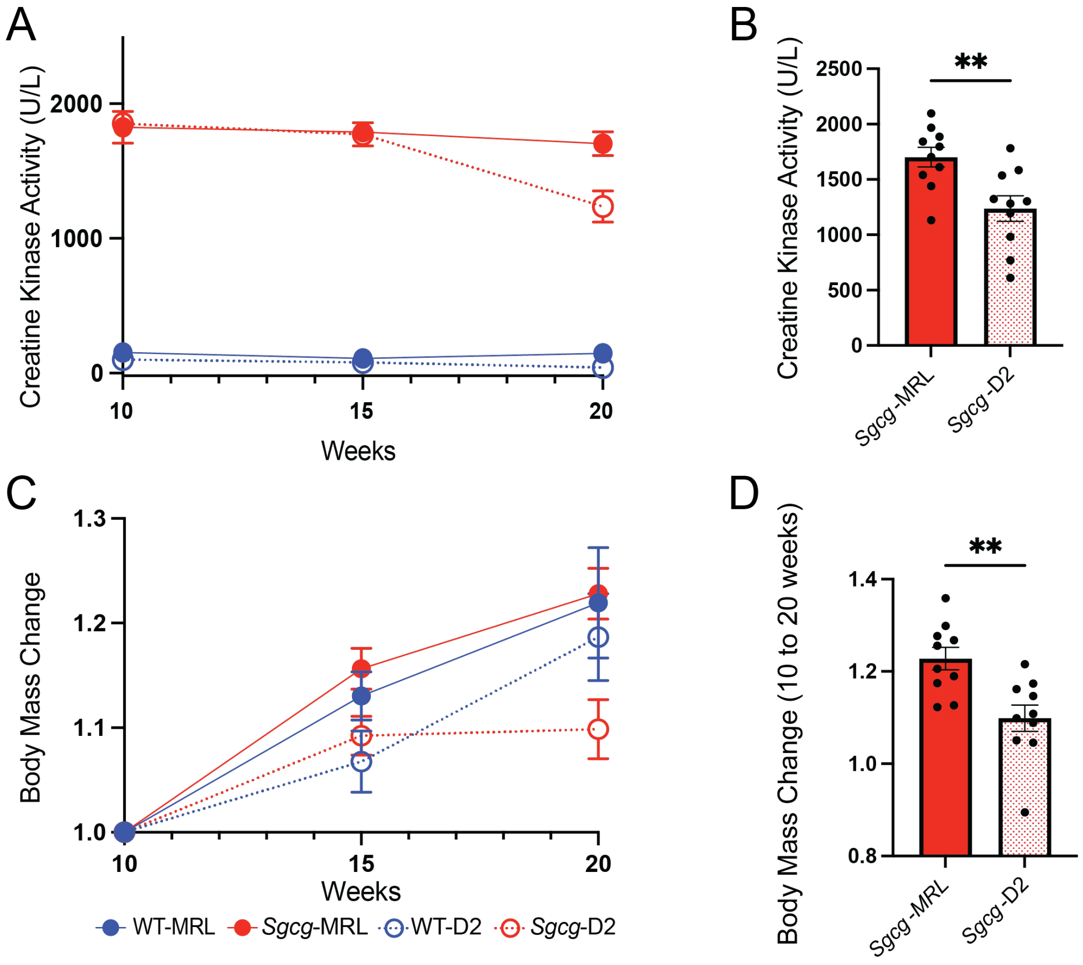

Supplemental Figure 1. Body mass and serum were collected and assessed from sex-matched cohorts at 10, 15 and 20 weeks. (**A**) Serum creatine kinase (CK) was monitored. Serum CK was elevated at all timepoints in *Sgcg*-MRL and *Sgcg*-D2 mice compared to their WT counterparts. (**B**) Serum CK in *Sgcg*-D2 mice declined at 20 weeks, paralleling the relative decrease in body mass and muscle mass observed in this cohort. (**C**) Change in body mass relative to the 10-week timepoint was recorded and plotted at 15 and 20-week timepoints. (**D**) The proportional body mass change from 10 to 20 weeks was increased in the MRL background. Graphical quantification of mean ± SEM. Student's t-test was used to determine statistical significance. ** p<0.01.

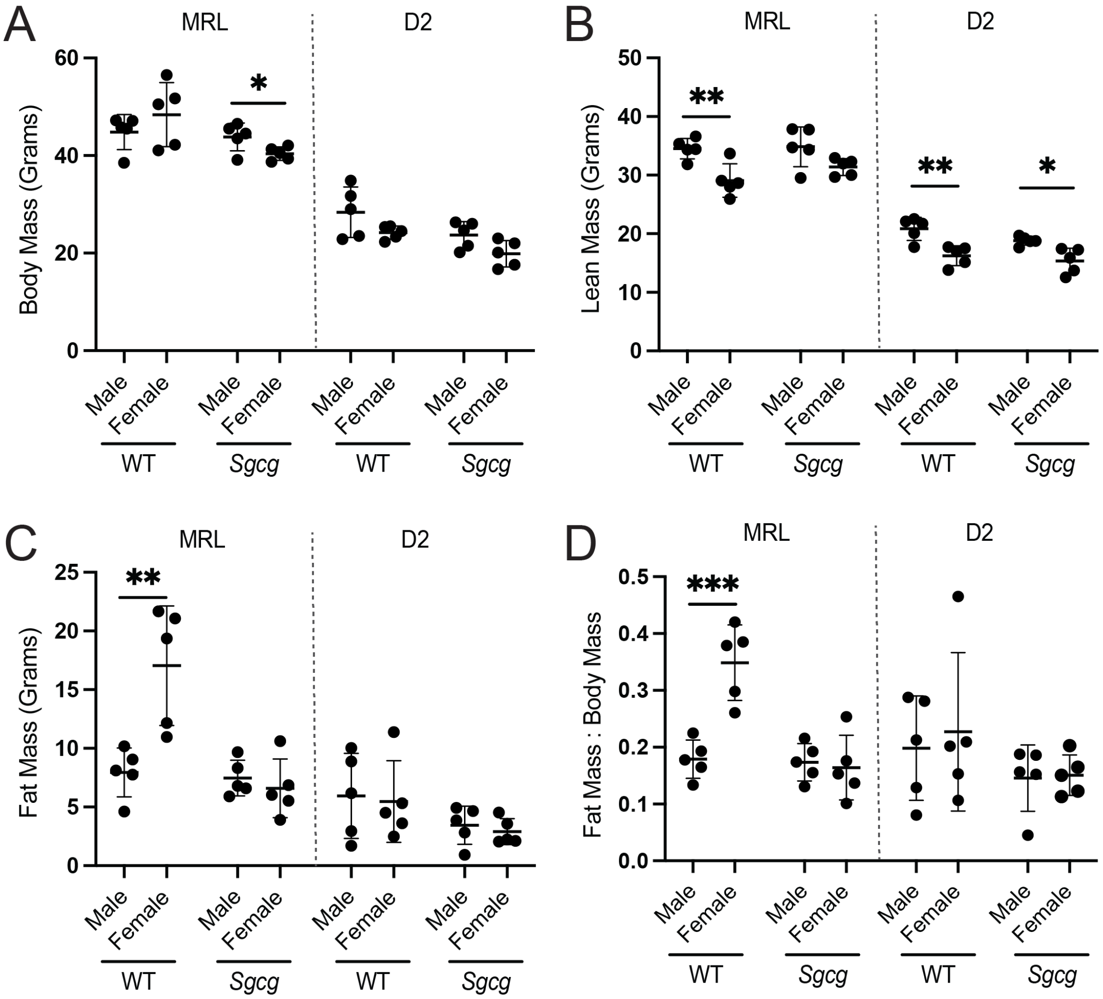

Supplemental Figure 2. MRL mice were larger than DBA/2J (D2) mice. Whole body mass and body mass composition analysis is shown from 5 male and 5 female mice at 20 weeks. (**A**) Body mass of the MRL strain was significantly higher than the D2 strain, and female *Sgcg*-MRL were lighter than their *Sgcg*-MRL male mice. (**B**) Lean mass was less in MRL-WT female mice compared to MRL-WT male mice while in Sgcg-MRL, male and female lean mass was similar. In the D2 background, females were smaller than males for both WT and Sgcg mice. (**C**) Fat mass of the WT-MRL female mice was significantly greater than WT-MRL male mice. (**D**) Fat Mass: Body Mass ratio was significantly greater in the females than the males for WT-MRL mice, and was highly variable for the other comparisons. Graphical quantification of mean ± SD. Student's t-test was used was used to determine statistical significance. * $p<0.05$, ** $p<0.01$, *** $p<0.001$, **** $p<0.0001$.

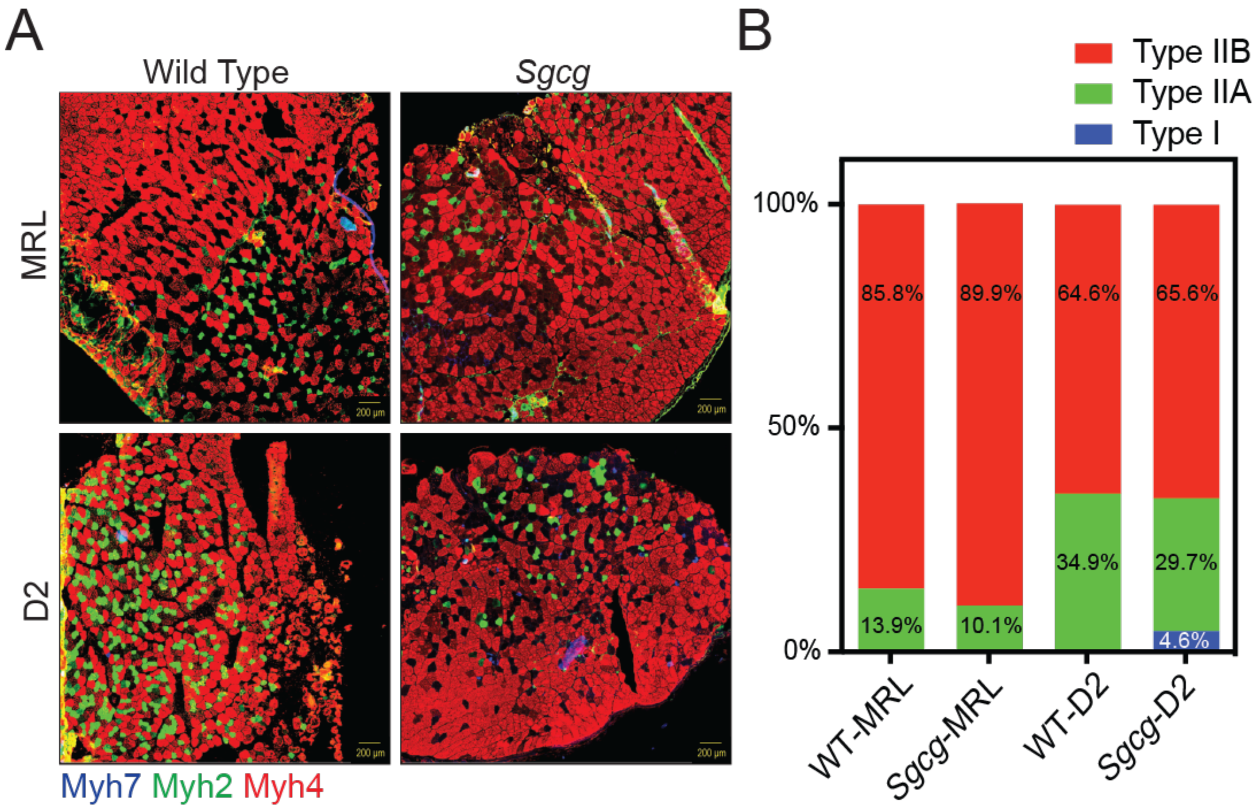

Supplemental Figure 3. Type I fibers in *Sgcg*-D2 mice were suppressed by the MRL background. *Tibialis anterior* (TA) muscles were harvested from 3 male mice, cryosectioned, and co-stained with antibodies to Myh7 (type I), Myh2 (type IIA) and Myh4 (type IIB). (**A**) Representative images of muscles showed a higher proportion of Myh4 (type IIB) fibers in the MRL background and a small percentage of Myh7 (type I) fibers in the *Sgcg*-D2 mice. (**B**) Overall, theh MRL background had greater Myh4 (type IIB) and less Myh2 (type IIA) compared to the D2 background.

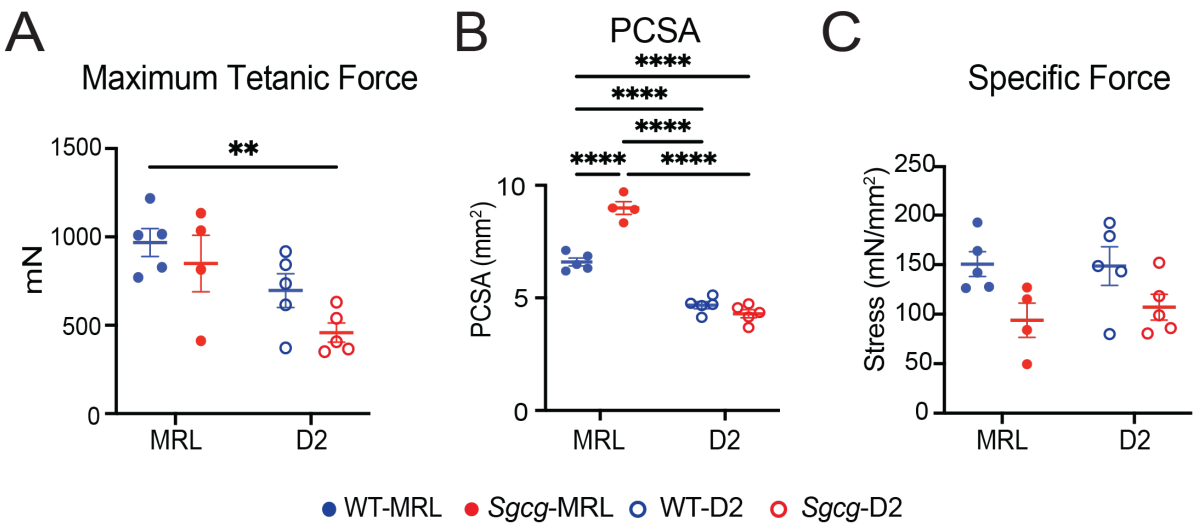

Supplemental Figure 4. Muscle force measurements demonstrate high variability and no significant differences in force production in *Sgcg* mice from either background strain. Muscle force mechanics were conducted on five female mice, 20 weeks of age. (**A**) The maximum tetanic force in the MRL background trended higher than D2 but did not reach significance. (**B**) Physiological Cross-Sectional Area (PCSA) of the MRL background was significantly larger. **C**. Specific force did not differ by strain or genotype. Graphical quantification of mean ± SD. Two-way ANOVA was used to determine statistical significance. * p<0.05, ** p<0.01, *** p<0.001, **** p< 0.0001.

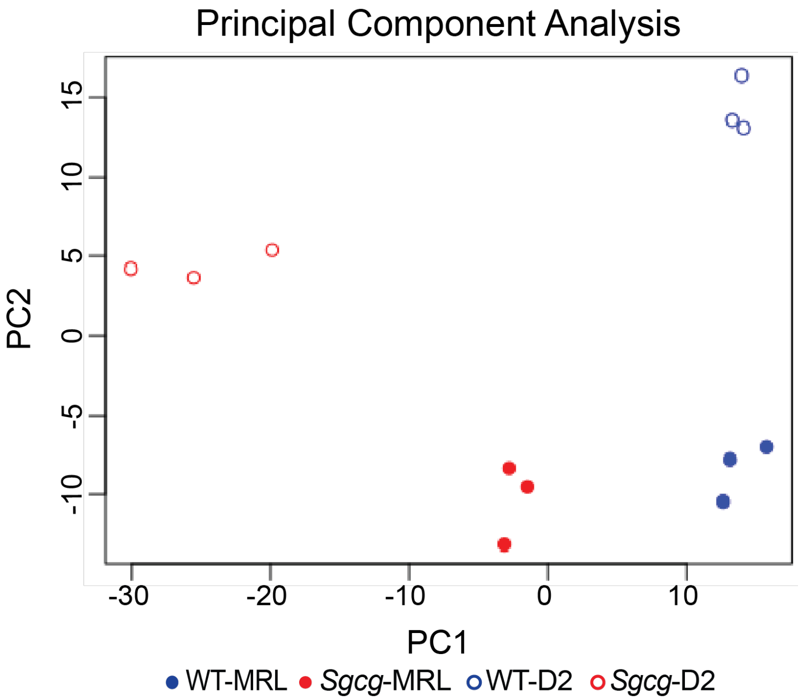

Supplemental Figure 5. Principal component analysis (PCA) of RNA sequencing shows distinct clustering of phenotypic cohorts.

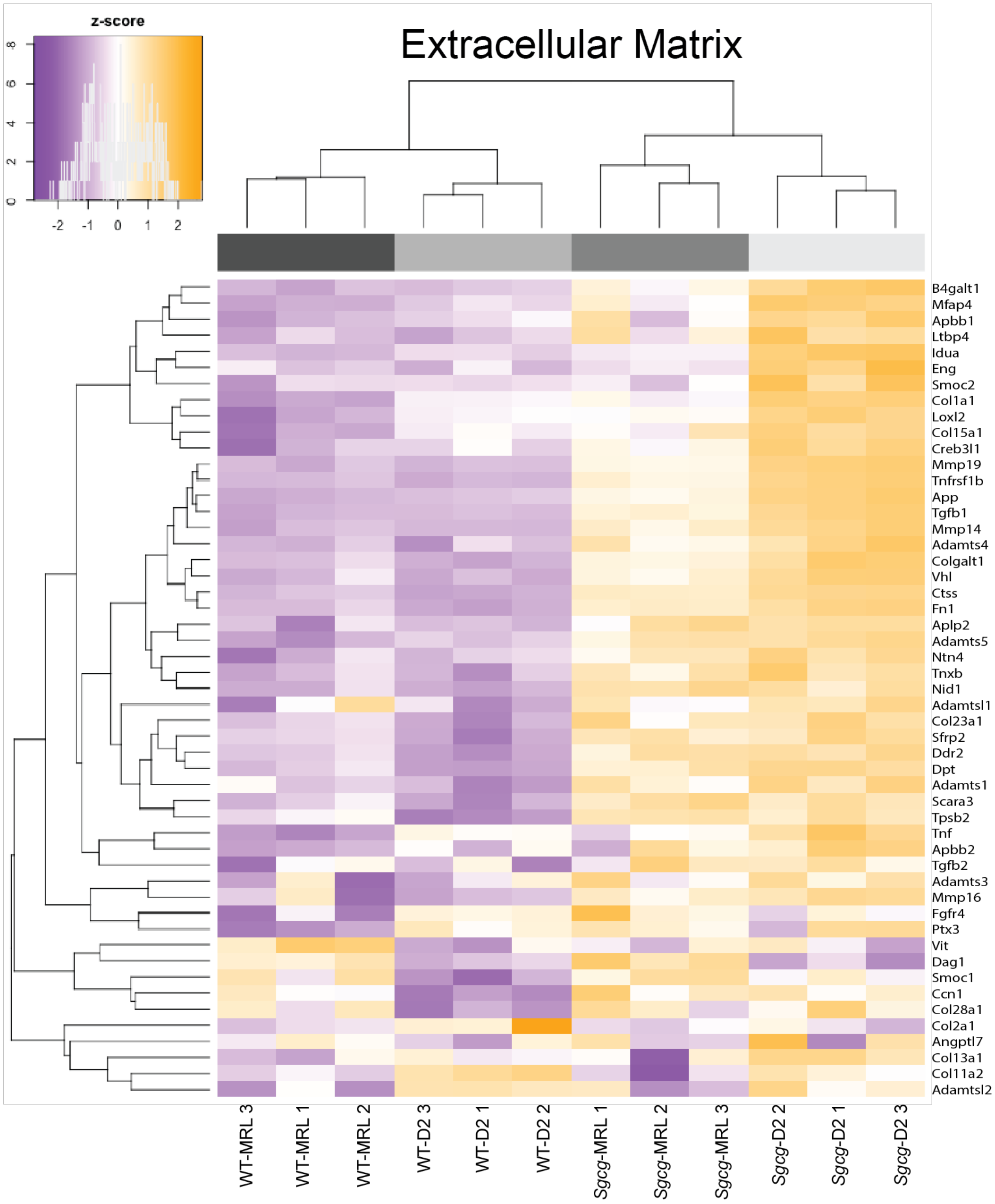

Supplemental Figure 6. Clustered heatmap indicates upregulation of ECM genes in the *Sgcg*-D2 muscle.

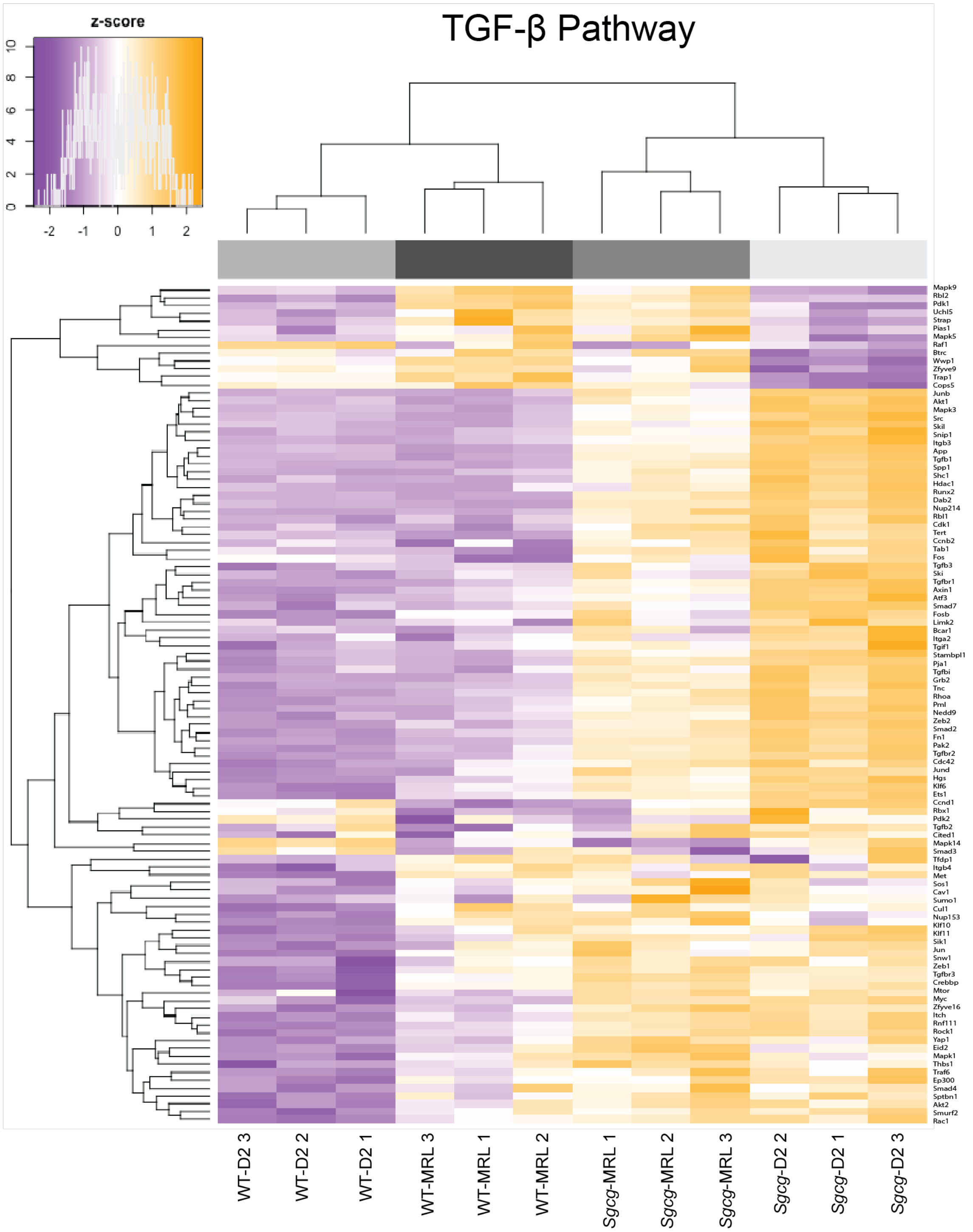

Supplemental Figure 7. Clustered heatmap illustrating the differential regulation of TGF-β genes in *Sgcg*-MRL compared to *Sgcg*-D2

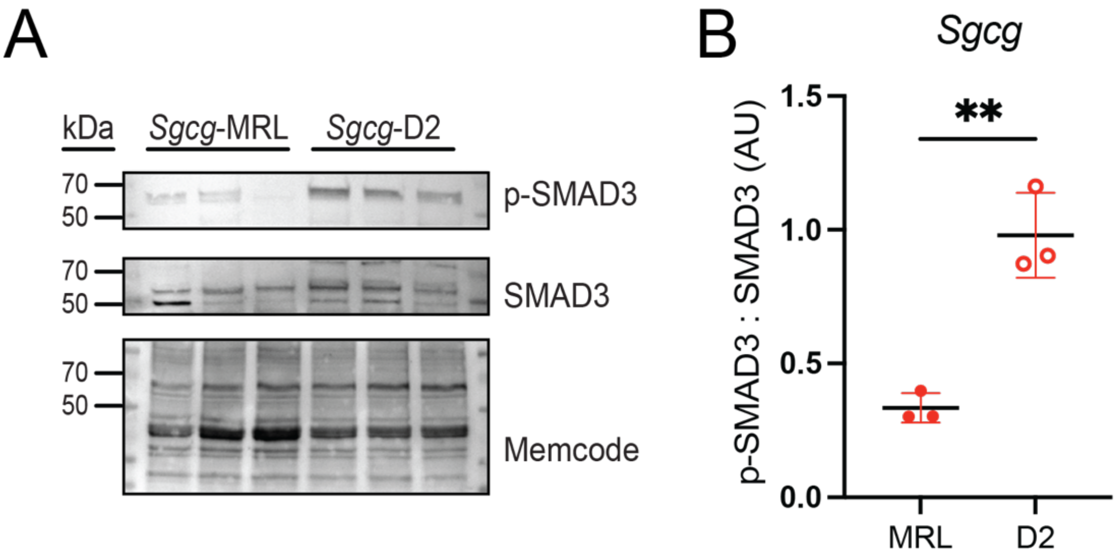

Supplemental Figure 8. (**A**) Immunoblotting of the gastrocnemis/soleus muscles from *Sgcg*-MRL and *Sgcg*-D2 mice showed reduction of phosphorylated SMAD3 (p-SMAD3) relative to total SMAD3 in *Sgcg*-MRL. (**B**) The ratio of p-SMAD3 to total SMAD3 was quantified and indicated TGF-β signaling was decreased in the MRL background. Graphical quantification of mean ± SD. Student's t-test was used to determine statistical significance. ** $p<0.01$.

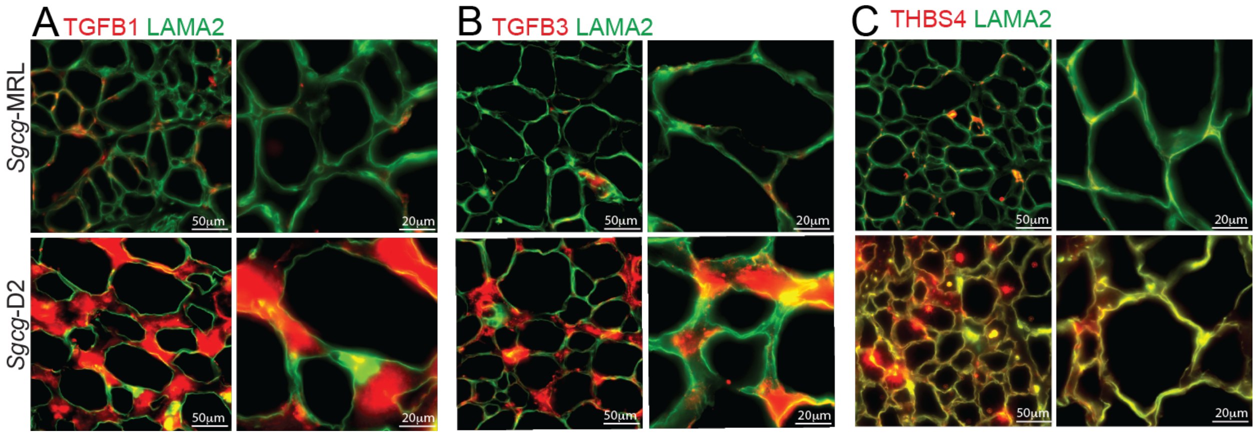

Supplemental Figure 9. Decellularized myoscaffolds were generated from *Sgcg*-MRL (top) and *Sgcg*-D2 (bottom) gastrocnemis/soleus (G/S) muscles. (**A**) Representative IFM images of dECM myoscaffolds were co-stained with TGF-β1 (red) and LAMA2 (green) antibodies. TGFβ-1 expression was demonstrably reduced in the *Sgcg*-MRL matrix, following the same pattern observed in the quadriceps. (**B**) G/S dECM myoscaffolds were co-stained with TGF-β3 (red) and LAMA2 (green) antibodies and showed reduced TGF-β3 expression in the *Sgcg*-MRL background. (**C**) Immunofluorescent images of thrombospondin-4 (THBS4, red) and LAMA2 (green) show marked reduction of THBS4 in the *Sgcg*-MRL matrix.

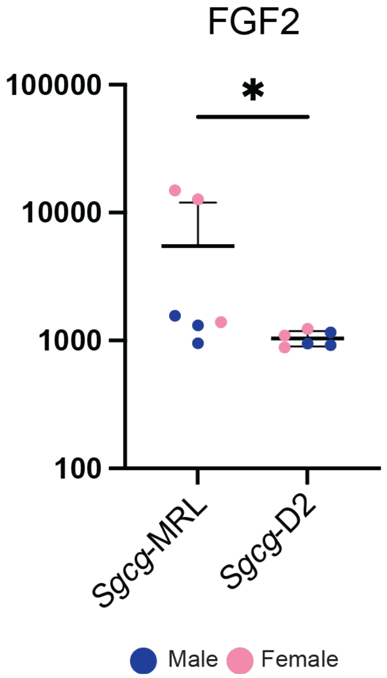

Supplemental Figure 10. Aptamer analysis of FGF2 serum indicated variability across animals, with exponentially higher expression in two *Sgcg*-MRL female mice. Statistical significance was determined using the Kolmogorov-Smirnov test. * $p<0.05$

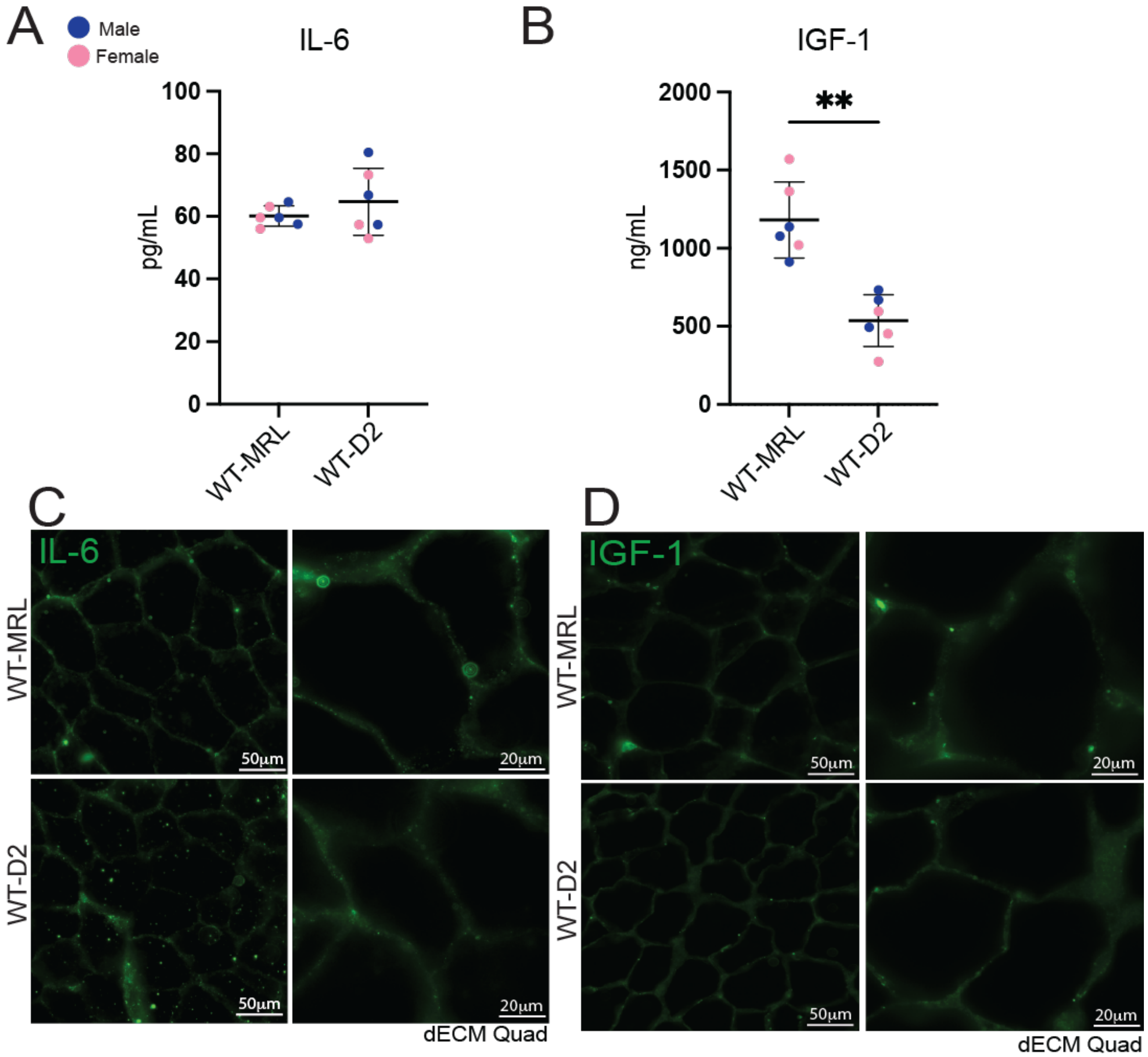

Supplemental Figure 11. ELISA of serum from WT-MRL and D2-WT mice at 20 weeks of age. (**A**) Serum IL-6 circulation was similar between WT-MRL and WT-D2 mice. (**B**) Serum IGF-1 was greater in WT-MRL strain compared to WT-D2 mice, which was the same patteren found in the dystrophic models (**Figure 6**). In contrast to what was seen in dystrophic dECMs, dECMs from WT-MRL and WT-D2 muscles showed minimal deposition of IL-6 (**C**) and IGF-1 (**D**), in contrast to the IL-6 and IGF-1 deposition seen in *Sgcg*-MRL muscle (**Figure 6**). Graphical quantification of mean ± SD. Statistical significance was determined using the Kolmogorov-Smirnov test. ** p<0.01.
